# Targeting the apoptotic Mcl-1-PUMA interface with a dual-acting compound

**DOI:** 10.18632/oncotarget.17294

**Published:** 2017-04-20

**Authors:** Jiyuan Liu, Zhen Tian, Nan Zhou, Xueying Liu, Chenyi Liao, Beilei Lei, Jianing Li, Shengyong Zhang, Hui Chen

**Affiliations:** ^1^ Department of Medicinal Chemistry, School of Pharmacy, Fourth Military Medical University, Xi’an 710032, Shaanxi, China; ^2^ Department of Chemistry, University of Vermont, Burlington, VT 05405, USA; ^3^ Northwest A&F University, Yangling, 712100, Shaanxi, China

**Keywords:** protein-protein interaction, Mcl-1 inhibitor, PUMA modulator, drug development, pharmacophore modelling

## Abstract

Despite intensive efforts in the search for small molecules with anti-cancer activity, it remains challenging to achieve both high effectiveness and safety, since many agents lack the selectivity to only act on cancer cells. The interface of two apoptotic proteins, myeloid cell leukemia-1 (Mcl-1) and p53 upregulated modulator of apoptosis (PUMA), has been recently affirmed as a target for treating cancers, as the disruption of Mcl-1-PUMA binding can reduce cancer cell survival and protect normal cells from apoptosis. However, therapeutic agents that target this interface are yet to be found. In this work, we combined pharmacophore modelling and biological tests to seek small molecules which target the Mcl-1-PUMA interface. For the first time, a small-molecule compound was identified. Its dual activity has been validated to reduce PUMA-dependent apoptosis while deactivating Mcl-1-mediated anti-apoptosis in cancer cells. Our results would provide a new avenue for the development of effective and safe anti-cancer agents.

## INTRODUCTION

Apoptosis is a critical cellular process in maintaining normal tissue homeostasis [1]. Proliferating cells in the absence of apoptosis is considered as an initial hallmark of cancer [2, 3]. As a mediator of the mitochondrial apoptotic pathway [4], p53 upregulated modulator of apoptosis (PUMA) is known to result in the displacement and activation of Bax/Bak through its binding to anti-apoptotic Bcl-2 proteins, leading to mitochondrial dysfunction and caspase activation, hence initiating apoptosis [5]. Absence of PUMA could efficiently protect normal cells (including thymocyte and lymphocyte) from the apoptosis induced by γ-irradiation and DNA-damaging drugs [6]. In many cancer cells, PUMA-dependent apoptosis is typically blocked by the elevated expression of myeloid cell leukemia-1 (Mcl-1, one of the most frequently overexpressed anti-apoptotic proteins) and the resulting deregulation of the Mcl-1-PUMA interaction [7]. While activation of Mcl-1 leads to survival of cancer cells, inhibition of Mcl-1 has been found to eliminate a series of cancer cells [8–11]. Therefore, small-molecule inhibitors of Mcl-1 can be useful as potential therapeutic agents for cancer treatments [10, 12, 13]. However, many Mcl-1 inhibitors obtained from prior efforts are potent to activate the mitochondrial apoptotic pathway by upregulating PUMA [10, 14], leading to apoptosis in both cancer and normal cells. The PUMA-dependent apoptosis of non-cancerous cells is a common side-effect of major agents used in current cancer therapy [15]. Therefore, it is challenging but rewarding to develop anti-cancer agents of high effectiveness and safety. As mentioned above, the antagonism between Mcl-1 and PUMA is vital to the survival of cells [16, 17]. When PUMA is upregulated, cells tend to apoptosis, on the contrary, when Mcl-1 is upregulated, cells tend to survival. The high-affinity interaction between Mcl-1 and PUMA has been verified by series of studies including yeast two-hybrid assay, co-immunoprecipitation studies and structural studies [16]. PUMA is reported to bind as an amphipathic helix in a deep hydrophobic groove on the surface of Mcl-1 through its BH3 domain [18, 19]. Targeting the druggable interface of Mcl-1-PUMA is promising to discover dual-acting compounds that could disrupt the interaction between Mcl-1 and PUMA, providing a way to settle the conflicts between effectiveness and side-effects of anti-cancer drugs [13, 20, 21].

**Scheme 1:**
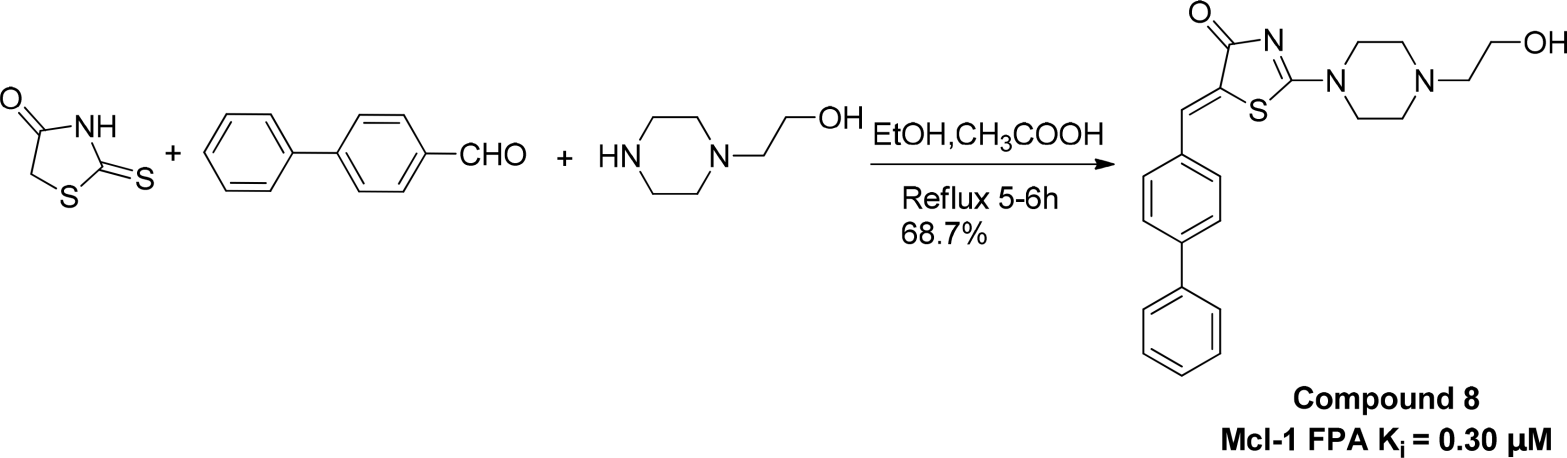
Preparation of compound 8

In this work, we for the first time used a combination of computer modelling and biological assays, to investigate the fundamental Mcl-1-PUMA interactions and to seek potential small molecules with high anti-cancer activity and safety. While some inhibitors of anti-apoptotic proteins (like ABT-263 and ABT-199) show promise in clinical trials for multiple cancers including acute myeloid leukemia and small-cell lung cancer [22, 23], our compound 8 (Comp8, Scheme 1) distinguishes itself in its dual activity to both suppress cancer cells and inhibit PUMA-dependent apoptosis of non-cancerous cells. This work not only offers validation for targeting Mcl-1-PUMA interface as a reliable strategy to develop novel anti-cancer drugs with both pronounced efficacy against cancers and low side effect in normal tissues, but also serves as a probe to achieve safer cancer therapies by modulating Mcl-1-PUMA interaction.

## RESULTS AND DISCUSSION

**Figure 1:**
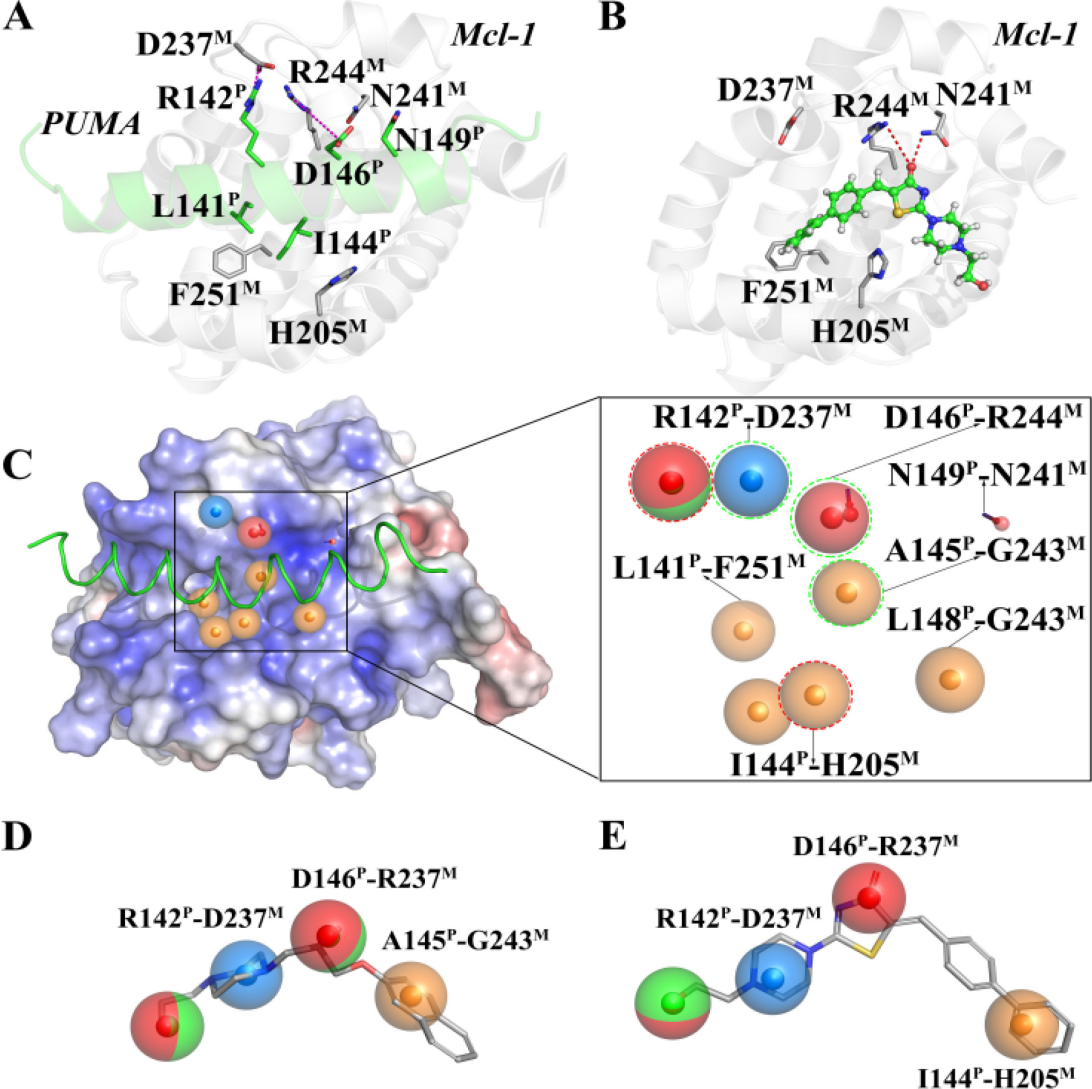
Key interactions at the interfaces of (**A**) Mcl-1-PUMA and (**B**) Mcl-1-Comp8 complexes. (**C**) The structure-based pharmacophore model (Left); The pharmacophore prototype merging (C) and (D) (Right). (**D**) The best ligand-based pharmacophore model. (**E**) The final pharmacophore model for prospective virtual screening. Details are present in the Supplementary Information.

The starting point for our study is the determination of compounds that potentially act on the Mcl-1-PUMA interface, using pharmacophore-based virtual screening. Molecular dynamics (MD) simulations of a 50 ns timescale were performed with the Mcl-1-PUMA complex models to provide the energetics and plasticity details of the interface. Using structural data (revealed by pairwise per residue free energy decomposition method in Supplementary Table 1) of the MD representative structure of Mcl-1-PUMA complex (Figure 1A) and the common features (revealed by multi-conformational alignment method) of the eight PUMA modulators (Supplementary Figure 2), we constructed a structure-based (Figure 1C left) and ligand-based (Figure 1D) pharmacophore model, respectively. A prototype of the combined pharmacophore model was further developed (Figure 1C right) by merging the two models. To improve the combined prototype for high sensitivity and specificity, the combined model was finally optimized to one hydrophobic feature (I144^PUMA^-H205^Mcl-1^) in the structure-based model, two hydrogen bond features (hydrogen bond acceptor and hydrogen bond donor) in the ligand-based model, and three shared pharmacophore features (R142^PUMA^-D237^Mcl-1^, D146^PUMA^-R244^Mcl-1^ and A145^PUMA^-G243^Mcl-1^) of the two models. Compounds constitute the SPECS database, were queried by the final pharmacophore model (Figure 1E). The eight highest-scoring compounds (namely Comp 1–8, see Supplementary Table 3) were directly purchased from SPECS and prepared for further biological tests.

**Figure 2:**
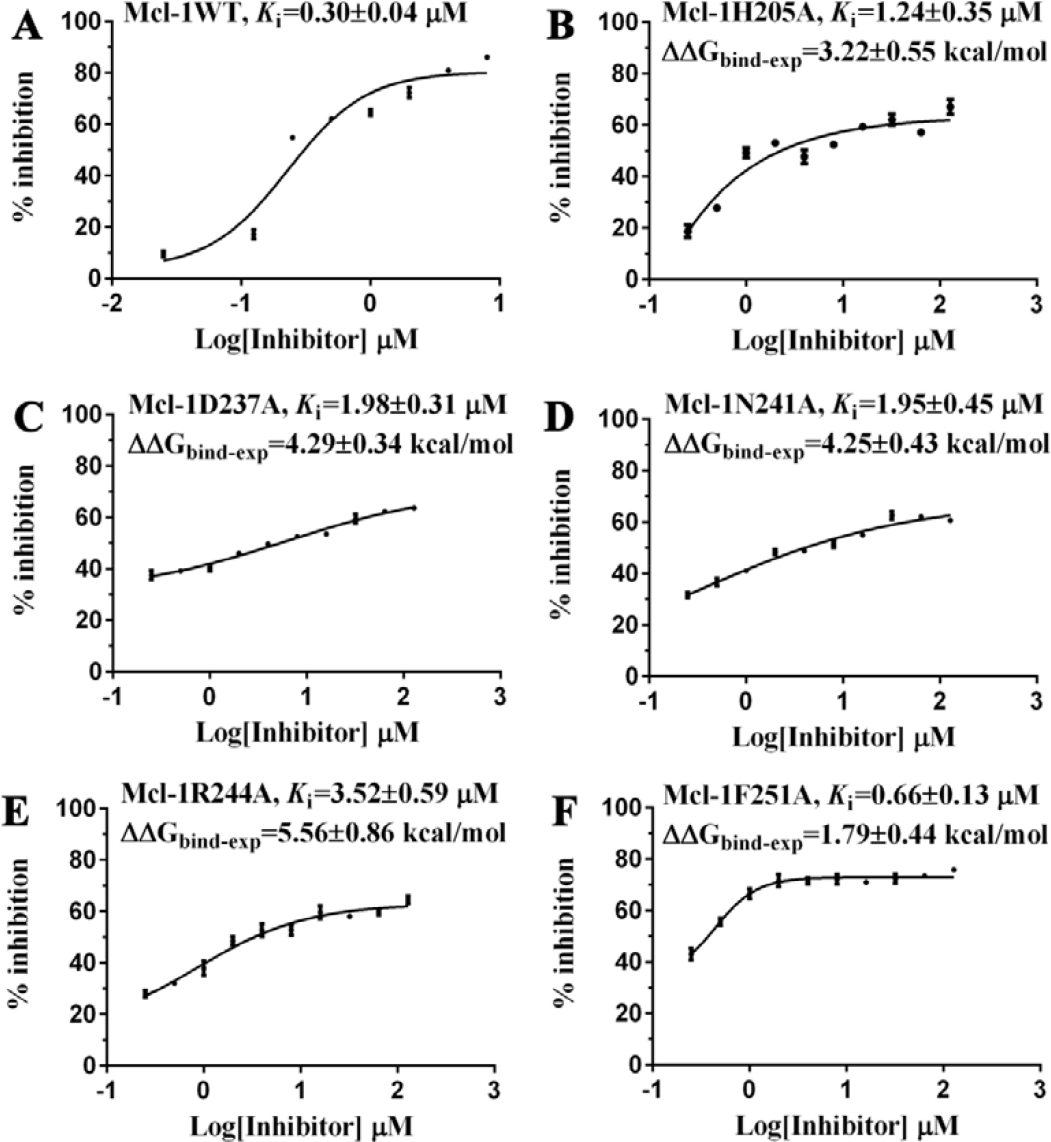
The inhibitory effects of Comp8 on (**A**) wild and (**B**–**F**) mutant types of Mcl-1. Details are present in the Supplementary Information.

**Figure 3:**
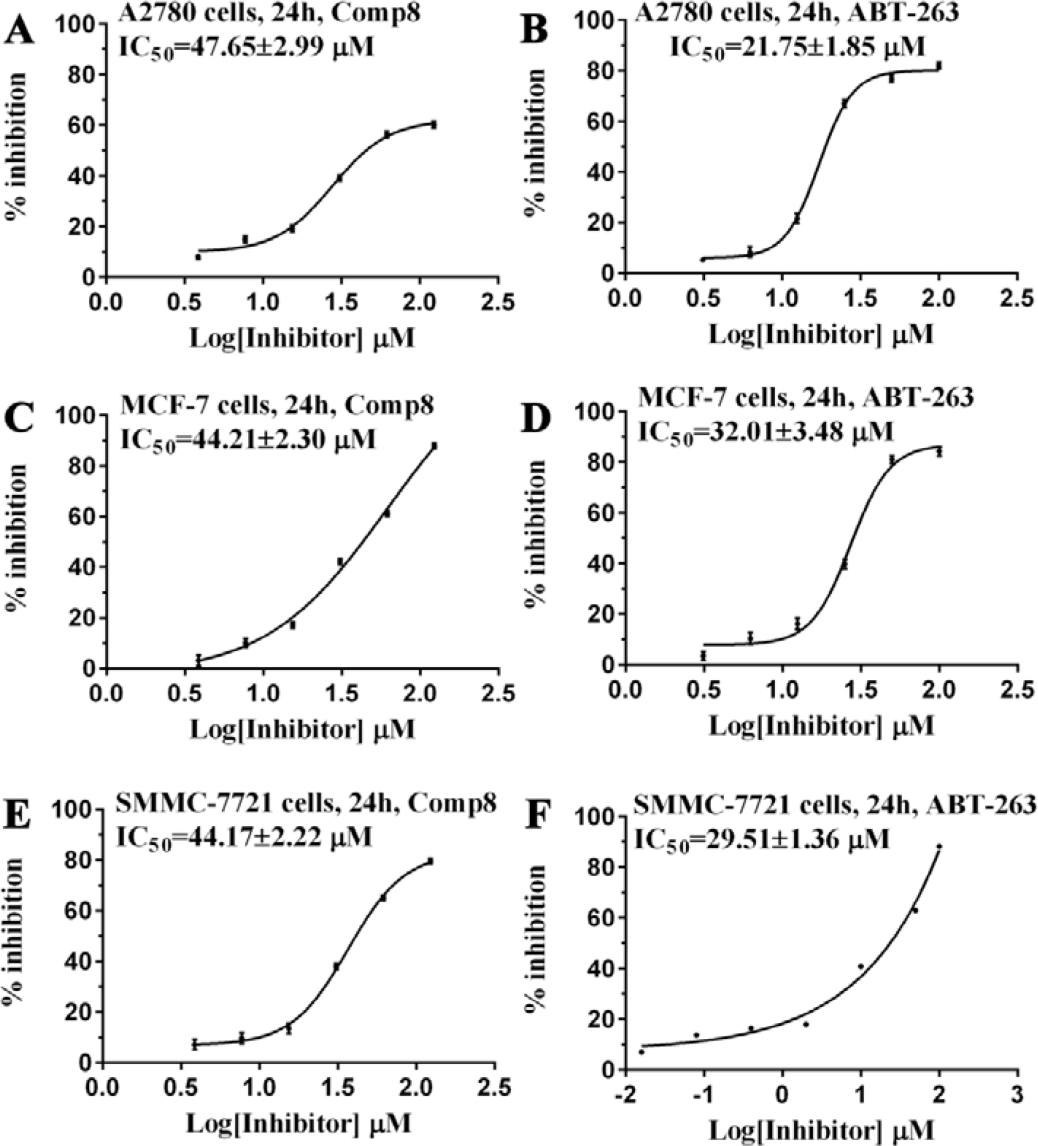
Comparison of (**A**, **C**, **E**) Comp8 and (**B**, **D**, **F**) ABT-263 on the activity to induce the apoptosis of three cancer cell lines.

Given the crucial role of R244^Mcl-1^ in developing Mcl-1 inhibitors [12, 19, 24], we firstly tested the inhibitory effects of these eight compounds on Mcl-1 by FPA competition assay. As shown in Figure 2A, Mcl-1 is significantly deactivated by Comp8 (*K*_i_ = 0.30 ± 0.04 μM). The *K*_i_ value is comparable with or lower than the counterparts of many other known Mcl-1 inhibitors (like UMI-77, *K*_i_ = 0.49 μM) [12, 19, 25, 26]. As for other seven compounds, they shed little effect on inhibiting Mcl-1 (Supplementary Figure 3). In the stable complex (Figure 1B) obtained by docking Comp8 into the binding center of Mcl-1 (Supplementary Figure 4) [27], Comp8 binds within the BH3-binding groove of Mcl-1 and is anchored by two polar contacts formed between the carboxylate group (Comp8) and N241/R244 (Mcl-1). Moreover, van der Waals interactions provided by the residue H205^Mcl-1^ are pivotal to the stability of Mcl-1-Comp8 complex. The stable occupation of Comp8 in the PUMA-binding groove of Mcl-1 possibly suppresses the Mcl-1-dependent anti-apoptosis of multiple cancer cells. To verify this, Comp8 was tested alongside a known BH3 mimetic ABT-263 for its activity to induce apoptosis in three cancer cell lines (A2780, MCF-7, SMMC-7721). These are well-known cancer cells that depend on Mcl-1 for continuous survival. As shown, on A2780, MCF-7 and SMMC-7721, the inhibitory effect of Comp8 is comparable with the counterpart of ABT-263 (IC50 in the range between 20 to 50 μM, see Figure 3) which is currently under clinical trials in numerous malignancies [28, 29]. Thereafter, flow cytometry detection (FCM) and western blot (WB) analysis of three apoptotic marker proteins (Caspase-3, Cyto-c and Bcl-2) were performed. According to the results of FCM, a substantial increase of apoptotic cells was observed in cancer cells treated with Comp8 (Supplementary Figure 5). WB analysis also revealed similar results (Supplementary Figure 6), the changes of three apotosis indicators fitted well with the characteristics of apoptosis. All the results suggested that Comp8 inhibited cancer cells by promoting apoptosis. The consistency of FPA competition assays and cell tests suggests that Comp8 can be developed as a potential lead compound against cancer through inhibition of Mcl-1.

By overlaying the two representative structures of Mcl-1-PUMA and Mcl-1-Comp8 complexes from MD simulations (Supplementary Figure 7), we find that Mcl-1 residues involved in the interactions with PUMA and Comp8 are greatly overlapped [18]. Like the hydrogen bond formed between R244 and the carboxylate group of Comp8, the same R244 forms a hydrogen bond with a conserved Asp of BH3 domain derived from BH3-only Bcl-2 proteins (PUMA as a representative) as well [12, 18].

**Figure 4:**
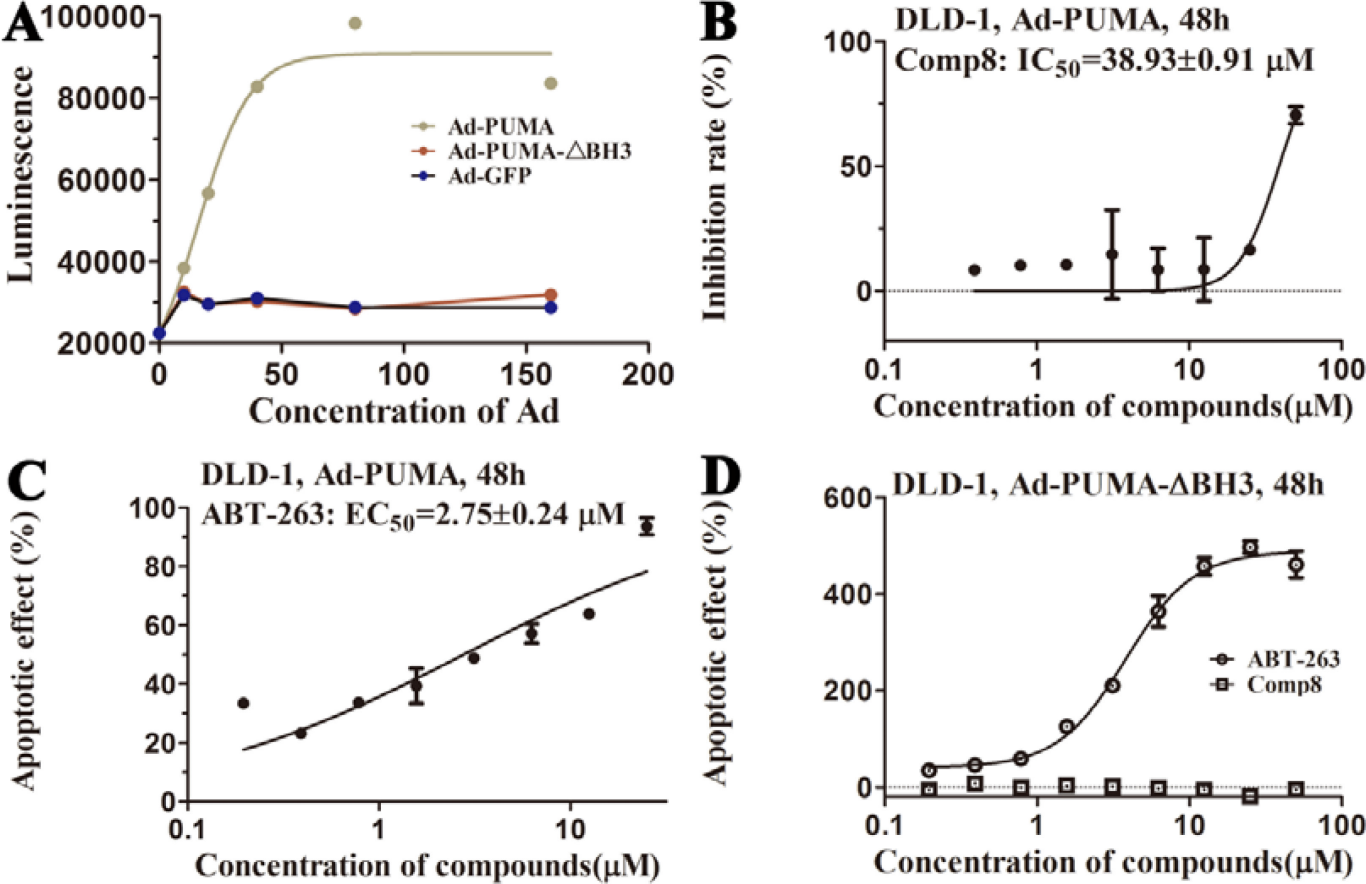
(**A**) Apoptosis is induced by PUMA with BH3 domain. The action curves of (**B**) Comp8 and (**C**) ABT-263 on apoptosis. (**D**) The effects of ABT-263 and Comp8 on DLD-1 cells infected with Ad-PUMA-ΔBH3. PUMA-dependent apoptosis can be effectively blocked by Comp8 which selectively targeting PUMA BH3.

Before testing the activity of Comp8 to disrupt the Mcl-1-PUMA interaction, it is necessary to determine whether these overlapped residues are key sites. Five Mcl-1 residues (H205, N241, R244, F251 and D237) which contribute significantly to Comp8/PUMA binding to Mcl-1 are subjected to site-directed mutagenesis (Supplementary Table 1). As revealed by FPA assays, the affinity between Mcl-1 and Comp8 is decreased at varying degree due to the mutation of each residue (Figure 2), while such mutations shed little effects on the binding of the fluorescence probe we used in FPA (Supplementary Table 4). Thereinto, D237, N241 and R244 are regarded as key residues in the Mcl-1-Comp8 interaction with the experimental binding free energy changes (ΔΔG_bind-exp_, Figure 2) beyond the threshold of hot-spot (> 4.0 kcal/mol) [30]. Likewise, these residues also contribute to the stabilization of the Mcl-1-PUMA complex [18]. This great overlap of key residues in Mcl-1-Comp8 and Mcl-1-PUMA complexes supports the postulation that Comp8 can disrupt Mcl-1-PUMA interaction. The overexpression of PUMA can cause severe death of DLD-1 cells (Figure 4A) [31]. However, the PUMA-dependent apoptosis is significantly inhibited by Comp8 (IC50= 38.93 ± 0.91 μM) (Figure 4B), which suggests its high selectivity to target the BH3 domain of PUMA (Figure 4D). In our tests, normal cells were fairly sensitive to the upregulation of PUMA, HUVECs infected with Ad-PUMA (Adeovirus overexpressing PUMA) exhibted rapid apoptosis. Even so, the addition of low-dose Comp8 (less than 12.5 μM) still shed inhibitory effect on the PUMA-induced apoptosis of HUVECs (Supplementary Figure 8).

In summary, we have identified a dual-acting compound, Comp8, at the same time to reduce multiple cancer cells and to inhibit PUMA-mediated apoptosis. Despite the comparable activity with most previously reported compounds in Mcl-1 inhibition [12, 19, 24], Comp8 displays crucial, unique activity to also inhibit PUMA-dependent apoptosis. This work represents the first study that fully considers such dual activity in the discovery of potential anti-cancer compounds. Moreover, apart from R244, the binding site of Comp8 is quite different from other Mcl-1 inhibitors [10, 19]. The discovery of dual-acting Comp8 sheds light on the discovery of new anti-cancer drugs as well as the improvement of current cancer treatments, especially when considering the PUMA-dependent apoptosis caused by γ-radiation and/or chemotherapeutic agents [32–35]. The convenient synthesis and purification of Comp8 (Scheme 1) together with its small molecular size further enhance its potential value in the the development of safer cancer therapeutics targeting Mcl-1-PUMA interface.

## MATERIALS AND METHODS

The Mcl-1-PUMA complex was retrieved from the crystal structure of the mouse Mcl-1 complexed with PUMA (PDB ID, 2ROC) [18]; The Mcl-1 complexed with compound 8 (Comp8) was constructed by molecular docking simulations using the program GOLD5.3 [27]; The molecular dynamic (MD) simulations for Mcl-1-PUMA and Mcl-1-Comp8 complexes were carried out by Amber12 package [36]; The final pharmacophore model for prospective virtual screening was designed in combination with the structure- and ligand-based pharmacophore modeling by using Ligandscout4.09 [37]; The pharmacophore-based virtual screening was performed using the Iscreen module provided by LigandScout4.09 [37], Only the compounds that matched all pharmacophore features were considered as a hit; The candidate compounds were subjected to FPA assay and cell tests to check their corresponding activities on disrupting Mcl-1-PUMA interface. All the details of experimental procedures were present in the Supplementary Information.

## SUPPLEMENTARY MATERIALS FIGURES AND TABLES




