# Supplementary material for: Targeting the apoptotic Mcl-1-PUMA interface with a dual-acting compound
Source: Oncotarget. 2017 Apr 20;8(33):54236–42. doi: 10.18632/oncotarget.17294 (PMC5589576; doi:10.18632/oncotarget.17294)
Supplement: Supplementary file 1 [file oncotarget-08-54236-s001.pdf]

Targeting the apoptotic Mcl-1-PUMA interface with a dual-acting compound

Supplementary Materials

RESULTS AND DISCUSSION

MD analysis of Mcl-1-PUMA and Mcl-1-Comp8 complexes

To reveal key interactions at the PUMA binding site, the Mcl-1-PUMA complex was performed with three individual 50 ns molecular dynamic (MD) simulations. As indicated by the small fluctuation of Cα root-mean-square deviation (RMSD) around 3.3 Å (Supplementary Figure 9A & C), the complex is highly stable during the entire course of simulations, showing several key contacts between PUMA and Mcl-1. Highlighted in Figure 1A, these contacts include L141^{PUMA}-F251^{Mcl-1}, R142^{PUMA}-D237^{Mcl-1}, R143^{PUMA}-D237^{Mcl-1}, I144^{PUMA}-H205^{Mcl-1}, A145^{PUMA}-G243^{Mcl-1}, D146^{PUMA}-R244^{Mcl-1}, D147^{PUMA}-H205^{Mcl-1}, L148^{PUMA}-G243^{Mcl-1} and N149^{PUMA}-N241^{Mcl-1}. Most of these contacts are non-polar interactions with a small contribution (ΔE_{pair} < 1.0 kcal/mol) to the overall stability of the complex, but two polar contacts R142^{PUMA}-D23^{Mcl-1} and D147^{PUMA}-H205^{Mcl-1} are crucial given their high interaction energies shown in our calculations (-9.43 and -3.22 kcal/mol respectively). Interestingly, the pairwise residues N149^{PUMA}-N241^{Mcl-1} contributed high favorable van der Waals energies but low electrostatic energies (Supplementary Table1), suggesting that the key interaction N149^{PUMA}-N241^{Mcl-1} could be exploited to increase the affinity by enhancing electrostatic energies.

To determine the protonation state of atoms in Comp8 (Supplementary Table2, Supplementary Figure 10), pKa and Microspecies plugins in Marvin software were used. the Mcl-1-Comp8 complex was constructed by docking. The complex formed by Mcl-1 and Comp8 was stable as well in the process of 50 ns MD analysis (Supplementary Figure 9B and 9D). By overlaying the MD representative structures of Mcl-1-PUMA and Mcl-1-Comp8 complexes, we detected that residues involved in the interactions between Mcl-1 and PUMA/Comp8 were partially overlapped. Moreover, the salt-bridge D237-R244 in Mcl-1 was disrupted by the formation of strong H-bonds between Comp8 and R244/N241. The distance between CG atom of D237 and CZ atom of R244 changed from 3.7 Å to 7.4 Å, the binding of Comp8 resulted in a 5.8 Å shift of D237. These all contributed to the disrupted interactions between Mcl-1 and PUMA resulted from the binding of Comp8.

Pharmacophore model construction

In the present study, the prototyping pharmacophore model (Figure 1C, the right part) was designed by merging the structure-based and ligand-based models. For the two models, the structure-based model (Figure 1C, the left part) was derived from the best MD representative structure of Mcl-1-PUMA complex (Figure 1A), or in other words, from the cluster 2 which exhibited the highest occupancy rate in the whole MD simulations (Supplementary Table5). Meanwhile, the ligand-based model (Figure 1D) was retrieved from multi-conformational alignment of 8 known PUMA modulators in Supplementary Figure 2 (excluding ZINC20719463 and ZINC20732753). To further refine the prototyping pharmacophore model shown in Figure 1C (the right part), the shared pharmacophore features located on three key interactions (R142^{PUMA}-D237^{Mcl-1}, D146^{PUMA}-R244^{Mcl-1} and A145^{PUMA}-G243^{Mcl-1}) were kept. Thereafter, for the 5 pharmacophore features located on 4 key interactions (L141^{PUMA}-F251^{Mcl-1}, I144^{PUMA}-H205^{Mcl-1}, D147^{PUMA}-H205^{Mcl-1}, L148^{PUMA}-G243^{Mcl-1} and N149^{PUMA}-N241^{Mcl-1}) in the structure-based part and 2 optional pharmacophore features (hydrogen bond acceptor [HBA] and hydrogen bond donor [HBD]) in the ligand-based part, the two hydrophobic features (L141^{PUMA}-F251^{Mcl-1} and L148^{PUMA}-G243^{Mcl-1}) were removed at first since these two pairwise residues contributed less than 1.0 kcal/mol ΔE_{pair} to the binding of PUMA (Supplementary Table1). The HBA feature (N149^{PUMA}-N241^{Mcl-1}) was not considerable either, since the pairwise residues N149^{PUMA}-N241^{Mcl-1} mainly contributed van der Waals energy instead of electrostatic energy (Supplementary Table1). Considering the repeated van der Waals interactions on the residue H205 (I144^{PUMA}-H205^{Mcl-1} and D147^{PUMA}-H205^{Mcl-1}), we finally reserved the hydrophobic feature on the right side of the pairwise residues I144^{PUMA}-H205^{Mcl-1}. Hence, the proposed pharmacophore models (Figure 1C, right) comprising three Shared features, two optional features (HBA and HBD) and one hydrophobic feature were selected for further refinement (removing features and setting features optional) by plotting ROC curves.

ROC analysis

To obtain the final pharmacophore model for prospective virtual screening, the proposed pharmacophore models were further validated and refined by plotting ROC curves. As shown in Figure 1E, the pharmacophore model retrieved enrichment factor (EF) values of 63.5, 17.8, and 8.9 at 1%, 5%, and 10% of the database prepared for validation, respectively. The high specificity is reflected by a total AUC value of 98% (Supplementary Figure 11). Therefore, we believe that it should be able to achieve high selectivity to identify Mcl-1-PUMA modulators and high specificity to eliminate false positives, which is suitable for prospective virtual screening.

Construction of Ad-PUMA virus

The qPCR analysis revealed that PUMA and PUMA-ΔBH3 are successfully overexpressed in infected DLD-1 cells (Supplementary Figure 12), representing the feasibility and reliability of following cell tests.

Synthesis of compound 8

Considering the potential value of Comp8 in suppressing PUMA-dependent apoptosis as well as overcoming ABT-263 resistance by targeting Mcl-1 for the treatment of cancer, we investigated the general procedures for the synthesis of Comp8. As illustrated in Scheme 1, Comp8 could be conveniently prepared by one-step. The structure of Comp8 was characterized by ^{1}H NMR, ^{13}C NMR and HRMS spectrum (Supplementary Figure 13). In addition, the crystal structure of Comp8 was determined by X-ray diffraction analyses (Supplementary Figure 13).

MATERIALS AND METHODS

Complex construction

The Mcl-1-PUMA complex was retrieved from the crystal structure of the mouse Mcl-1 complexed with PUMA (PDB ID, 2ROC) [1]. The Mcl-1 complexed with compound 8 (Comp8) was constructed by molecular docking simulations using the program GOLD5.3 [2]. Before docking, it is necessary to determine the pKa values of all proton receiving and donating atoms in Comp8 at first. The major protonation form of Comp8 at a specified pH was determined by the Microspecies plugin of Marvin software [3]. In order to mimic the natural experiment environment, the crystallographic structure of human Mcl-1 which bound to its own 16-mer BH3-peptide was selected as the receptor for the Comp8 docking simulations (PDB ID, 4HW4; resolution, 1.53 Å) [4]. The structure of human Mcl-1 was performed 5000 steps minimization in Amber12 with ff99SB force field [5, 6]. The 3D structure of Comp8 was derived from crystallographic structure generated by our chemistry experiment section (3.5 Synthesis of compound 8), and optimized 2000 steps by Amber12 using the GAFF force field prior to the docking simulations [7]. In order to determine the binding site of human Mcl-1 against Comp8, PocketQuery was employed to predict the promising starting points in the protein–protein interaction (PPI) interfaces with the two crystal structures of the Mcl-1 complexes [8]. The Chemplp score implemented in GOLD was employed to finely reproduce the best binding model of the complex since it was superior to other scoring functions in GOLD for pose prediction [9], we chose the best scored pose as the initial structure for molecular dynamics (MD) simulations. Based on the high amino acid sequence identity (88.54%) between mMcl-1 (mouse Mcl-1) and hMcl-1 (human Mcl-1), mMcl-1 and hMcl-1 were uniformly called Mcl-1 in our study, and residues of Mcl-1 were numbered after the residue sequence in crystal structure of mMcl-1 (Supplementary Figure 1). All the details of molecular docking simulations were performed according to our previous reports [10–12].

MD simulations and pairwise per-residue free energy decomposition

All the molecular dynamic (MD) simulations for Mcl-1-PUMA and Mcl-1-Comp8 complexes were carried out by Amber12 package [5]. After electrostatic potential calculations at the HF/6-31G* level using Gaussian 09 program package [13], the force filed parameters of the ligand Comp8 were analyzed by the GAFF method [14], and the partial atomic charges for the Comp8 atoms were calculated using the restrained electrostatic potential (RESP) fitting protocol [15]. The AMBER for bioorganic systems force field (ff99SB) was applied to depict the PUMA and Mcl-1 protein parameters [6]. The global charge of each system was neutralized by adding appropriate number of counterions. The two systems were solvated in a rectangular box of TIP3P water [16] with a minimum solute wall distance of 10 Å. For Mcl-1-PUMA and Mcl-1-Comp8 systems, we performed three individual 50 ns MD at different seeds for production phase without any restraint. MD results were analyzed with Ambertools13 package based on three individual 50 ns MD trajectories, and the data were averaged [5].

Experimental evidence suggested that the challenges of designing small-molecule protein-protein interactions modulators (PPIMs) can be overcome [17], especially for Bcl-2 family proteins [18]. Residues participating in important interactions have been shown to be spatially clustered in protein-protein interfaces, forming hot-spots regions [17]. In order to provide a possibility for the development of the small-molecule Mcl-1-PUMA interactions modulator and mimicking localized interactions at these hot spots, the protein-protein and protein-ligand interactions spectrums of each complex (Mcl-1-PUMA and Mcl-1- Comp8) were schemed based on pairwise per-residue free energy decomposition method [17, 19, 20] using the Molecular Mechanics-Poisson-Boltzmann Surface Area (MM-PBSA) [21] analysis by the mmpbsa.py module in AmberTools13 [22]. In order to guide the subsequent structure-based pharmacophore

modeling and to uncover the accurate binding mode for Mcl-1-Comp8 complex, we applied the average-linkage algorithm to perform clustering analysis (Supplementary Table 5) of the 50 ns MD simulation trajectories for these two systems. All the detail of MD simulations was performed according to our former reports [12, 23, 24].

Data sets preparation

For all data sets, OMEGA [25, 26] incorporated in LigandScout 4.09 [27] were employed to generate 3D multi-conformational databases.

PUMA modulators from literature

PUMA modulators for training sets were determined based on one previous report by Gabriela et al. (2011) [28]. In the report, 13 compounds were suggested to suppress PUMA-induced apoptosis at 25 μM [29]. The compound structures were checked in the original literature for correctness, and finally, 10 diverse structures (Supplementary Figure 2) were transformed into 3D conformational models using OMEGA [25, 26] in LigandScout4.09 [27], allowing BEST settings with a maximum of up to 500 conformers per molecule.

Decoy database

A decoy set was extracted from the DUD-E database [29, 30]. Based on the clustering of structurally diverse compounds using ECFP4 fingerprint, we selected 500 compounds as the final decoy database. For each decoy molecule, a maximum of 500 conformers was generated using OMEGA (Best settings) in the LigandScout 4.09.

Specs database for prospective virtual screening

The Specs database, version Jun2015_10mg (www.specs.net) including 210,265 entries was downloaded and calculated as a multi-conformational database.The 3D multi-conformational screening database were created using OMEGA [25, 26] incorporated in ligandscout4.09 [27]. For each molecule entry, a conformation model consisting of a maximum number of 100 conformers per molecule was computed with BEST settings so as to reproduce the flexibility of molecules during the prospective virtual screening. Finally, 210, 211 compounds were subjected to the prospective virtual screening conducted on the basis of pharmacophore modeling. The resulting virtual screening library was stored in the LigandScout database format (LDB).

Pharmacophore modeling and virtual screening

In this study, the final pharmacophore model for prospective virtual screening was designed in combination with the structure- and ligand-based pharmacophore modeling. Ligandscout4.09 [27] was employed for pharmacophore generation and refinement, pharmacophore features mapping and prospective virtual screening.

Based on the MD simulations, the cluster analysis of Mcl-1-PUMA complex and pairwise per-residue free energy decomposition of the Mcl-1 complexed with PUMA were performed. The structure-based pharmacophore model was derived from MD representative structure clusters (cluster exhibiting a high occupancy rate in the whole MD simulations) of Mcl-1-PUMA complex. Only the pharmacophore features representing the interactions of the PUMA BH3 domain in complex with Mcl-1 were selected to use in the subsequent pharmacophore modeling procedure.

The pharmacophore radial distribution function (RDF) was employed to cluster the training molecules according to their flexibility (number of conformations). The training molecules selected from reported PUMA modulators were loaded into the espresso module of Ligandscout to construct the ligand-based pharmacophore models. Only features present in all training molecules were considered for model building. During the ligand-based pharmacophore generation, the Relative Pharmacophore-Fit scored the number of matching pharmacophore features, and the root-mean-square deviation (RMSD) of the pharmacophore alignment normalized to 0~1. The final ligand-based model was selected with the highest Relative Pharmacophore-Fit score.

On the basis of feature-based alignments between the best structure- and ligand-based pharmacophore models, features overlapping too much were combined into a single one. The prototyping pharmacophore model was selected for further refinement (removing features, setting features optional) based on the spectrum of Mcl-1-PUMA pairwise per-residue free energy decomposition, and calculation of the Receiver Operating Characteristic (ROC) [31, 32] curves obtained by plotting our decoy database. In the ROC curves, we defined y axis as the TP rate (active compounds correctly classified/all active compounds), versus x axis as the FP rate (inactive compounds incorrectly classified as active/all inactive compounds). The point (0.1) represented a perfect classification. The area under the curve (AUC) varied between 0 and 1 [31, 32].

To search for potential Mcl-1-PUMA modulators from virtual screening library, the virtual screening based on the final pharmacophore model was performed using the Iscreen module provided by LigandScout4.09 [27]. Only the compounds that matched all pharmacophore features were considered as a hit. After visual inspection of the original hits list, compounds at the top rank of Pharmacophore-Fit score were selected for biological tests.

Inhibitory effects of candidate compounds on Mcl-1

Mcl-1 protein expression and purification The gene sequence encoding residues 172-327 Mcl-1 (GenBank accession: AF198614.1) was cloned into the pET-43.1a (+) vector (Novagen) to construct Mcl-1-expressing plasmid (Mcl-pET-43.1a), the constructed plasmid was checked by sequencing and transformed into the competent BL21 strains. To express soluble Mcl-1 protein, the BL21 strains containing Mcl-pET-43.1a were firstly grown to OD_{600}=0.6 in the condition of 37°C, 200 rpm. Thereafter, the Mcl-

1 protein was induced by 0.6 mM IPTG for 20 h in the condition of 20°C, 160 rpm. The strains were harvested by centrifugation (6000 rpm, 10 min, 4°C) and broken by ultrasonication (10 s operation and 10 s stop, 5 min). After cleared by centrifugation (12000 rpm, 30 min) and filtration (0.44 μm filter membrane), the lysate was purified by Ni^{2+}-NTA sepharose gel column (7Sea) following the manufacturer's instructions. The purified Mcl-1 was analyzed by 15% SDS-PAGE and dialyzed against 10 mM PBS (pH 7.4) before being stored under −80°C for later use.

FPA competition assays The FITC labelled BH3 peptide of Mcl-1 (FITC-Mcl-BH3, FITC-AHx-KALETLRRVGD GVQRNHETAF-NH2) was synthesized and used as fluorescence probe in the FPA competition assays [33]. All assays were performed in the assay buffer (pH 7.4) composed of 20 mM Tris, 50 mM NaCl, 3 mM DTT and 2% DMSO. The FPA competition assays were carried out in the 96-well, black, flat-bottom plates (Nunc). To check the interference effects of candidate compounds on the interaction between Mcl-1 and FITC-Mcl-BH3, solutions containing 250 nM Mcl-1 and 250 nM fluorescence probes were titrated with 250 μM selected compounds (0-128 μM, 2-times gradient) dissolved in the assay buffer. The emission fluorescence changes were recorded from 480 to 540 nm by TECAN Infinite M1000 Microplate Spectrophotometer with excitation wavelength being 432 nm. The IC_{50} calculations were performed by Graphpad Prism 5 (Graphpad software, Inc.).

Tests of candidate compounds on suppressing cancer cells

Cell lines including A2780, MCF-7 and SMMC-7721 were cultured to the logarithmic growth phase in 3 ml complete media (RPMI1640 plus 10%FBS), respectively. After adjusting their concentrations to 5×10^4 cells/ml with complete media, these cell lines were plated into 96-well, transparent, flat-bottom plates (150 μl/well), each point was prepared in three replicates. The density of cultured cell lines was evaluated by MTT tests. When the density reached 60%, tested compounds of different concentrations (diluted to 123.43 μM, 61.715 μM, 30.858 μM, 15.429 μM, 7.714 μM, 3.857 μM and 0 μM by complete media) were added to wells, each concentration was tested in three replicates. 24h later, each well was added 15μl MMT and cultured for 3-4h. Then absorb the liquid in each well thoroughly, add 200μl DMSO, and shake for 10 min at room temperature. Record the OD values of each well with TECAN Infinite M1000 Microplate Spectrophotometer (under 492 nm). Cell lines can be effectively suppressed by tested compounds were also subjected to ABT-263 tests, the procedures were similar to what described above. All the cells were cultured under the condition of 37°C, 5% CO2.

Flow cytometry

Tested cells (A2780, MCF-7 and SMMC-7721) were plated in 6-well plates (1×10^5 cells/well) and treated with/without the given concentration of Comp8 for 48h (47.42 μM for A2780 and MCF-7, 27.80 μM for SMMC-7721). All cells were detached by trypsin and washed (three times) by precooled PBS. The resuspended cells were stained by Annexin V-FITC and PI in sequence before being detected by flow cytometry.

Western blot analysis

A2780, MCF-7 and SMMC-7721 cells treated with and without Comp8 were harvested, washed by precooled PBS and split by RIPA lysis buffer (Thermo) containing PMSF (protease inhibitor). Quantified protein samples were separated by SDS-PAGE and then transferred to NC membranes. After being blocked for 1h, all membranes were incubated with primary antibodies against protein Caspase (1:500, Abcam), Cyto-c (1:500, Abcam) and Bcl-2 (1:500, Abcam) at 4℃ overnight. The membranes were then incubated with HRP-conjugated secondary antibodies (1:10000, Jackson) for 1 h at 37°C, the signals were detected with ECL western blot detection system (Boster).

Key residues discovery

In the present study, key residues involved in the interaction between Mcl-1 and Comp8 were revealed by using site-directed mutagenesis and FPA competition assays in combination. According to the results of pairwise per-residue free energy decomposition, the residues contributing a lot to the binding of PUMA/Comp8 to Mcl-1 were subjected to site-directed mutagenesis. By reference to the primers listed in Supplementary Table5, overlapping extension PCR was conducted to realize site-directed mutagenesis [33]. After verified by gene sequencing, the individually mutated PCR products were cloned into the expressing vector pET-43.1a (+). The acquisition of mutant Mcl-1 proteins (MT Mcl-1) was performed according to the expression and purification of wildtype Mcl-1 (WT Mcl-1) mentioned above. To measure the effects of site-directed mutations on the Mcl-1/ Comp8 interaction, the mutant types of Mcl-1 were subjected to FPA competition assay, and the corresponding K_i values were transformed into binding free energy changes ($\Delta\Delta G_{bind\text{-}exp}$) based on the following formula:

$$\Delta\Delta G_{bind\text{-}exp} = RT\ln(K_{i\text{-}MT}/K_{i\text{-}WT})$$

Here, $K_{i\text{-}MT}$ and $K_{i\text{-}WT}$ are K_i values between MT Mcl-1/WT Mcl-1 and Comp8, respectively. R is ideal gas constant and T is temperature in Kelvin.

Tests of candidate compounds on blocking PUMA-dependent apoptosis

Construction and validation of recombinant Adenovirus By taking pHBAd-MCMV-GFP (Hanbio) as vector, the construction and purification of Adenoviruses overexpressing PUMA and PUMA-ΔBH3 (PUMA without the BH3 domain) were performed according to former reports [35, 36]. Thereafter, to check whether the recombinant Adenovirus could result in overexpression of PUMA and PUMA-ΔBH3, the DLD-1 cells infected with Ad-PUMA (Adenovirus expressing PUMA) and Ad-PUMA-ΔBH3 (Adenovirus expressing PUMA-ΔBH3) were respectively subjected to qPCR analysis with GAPDH as internal standard [37].

Tests of PUMA BH3 domain on DLD-1 apoptosis

The prepared DLD-1 cells were infected with Ad-PUMA (Adenovirus expressing PUMA), Ad-PUMA-ΔBH3 (Adenovirus expressing PUMA without BH3 domain) and Ad-GFP (Adenovirus expressing GFP) of various multiplicity of infection (MOI, 6 points, from 0 to 160) in 96-well, white, flat-bottom plates (Nunc). 48 h later, replace 40 μl media with 40 μl apoptosis reagent from Caspase-Glo® 3/7 Assay kit (Promega) in each tested well and incubate for 1h. Shake gently and record the luminescence of each well on TECAN Infinite M1000 Microplate Spectrophotometer. The data were analyzed by Graphpad Prism and suitable MOI of Ad-PUMA for following cell infection can be determined in this process.

Tests of candidate compounds on DLD-1 apoptosis

100 μl DLD-1 cells suspension in logarithmic growth phase were cultured in 96-well, white, flat-bottom plates (Nunc) for 24 h before being infected by Ad-PUMA of suitable MOI. The infected DLD-1 cells were incubated for 6 h. Thereafter, 100 μl candidate compounds plus ABT-263 diluted in complete media (RPMI1640 media supplemented with 10% FBS) were added to make the final compound concentrations range from 0 to 50 μM (2-times gradient), 10 points were selected for each compound. The results were detected by Caspase-Glo® 3/7 Assay kit (Promega) following above descriptions.

Tests of candidate compounds on HUVECs apoptosis

HUVECs were plated in 96-well plates (1×10^5 cells/well) and infected by Ad-PUMA of suitable MOI. 6h later, add different concentrations of candidate compounds (0, 0.2, 0.39, 0.78, 1.56, 3.12, 6.25 and 12.5 μM). After 48h, check the fluorescence intensity of each well with Caspase-Glo®3/7 Assay Kit (Promega) and photograph using fluorescence microscope.

Throughout the cell tests, HUVECs and the colon cancer cell lines DLD-1 were cultured in the complete media and maintained in the conditions of 37°C, 100% RT and 5% CO2. Two replicates were performed for each point. All the data were analyzed by Graphpad Prism 5 (Graphpad software, Inc.).

Synthesis of compound 8

To a suspension of rhodanine (7.57 mmol) in dry ethanol (10 mL), 4-Biphentlaldehyde (11.3 mmol), N-(2-hydroxyethyl) piperazine (11.3 mmol), and glacial acetic acid (0.75 mmol) were added and the resulting mixture was further stirred under reflux for 5-6 h. The reaction was completed and then cooled in an ice bath. The precipitated crystalline solid was filtered off, washed with cooled ethanol and dried under vacuum. The crude product was subjected to silica gel column chromatography (DCM: methanol 9:1) to afford the desired compound as yellow solid.

Data for Comp8:Yield = 68.7%.^{1}H NMR(400MHz, $CDCl_3$) δ7.87(s, 1H,vinyl), δ7.70-7.61 (m, 6H,Ar) , δ7.50-7.40 (m, 3H, Ar), δ5.32（s, 1H, OH）,δ4.1(t, 2H, CH_2) , δ3.71 (m, 4H, NCH_2) , δ2.73 (t, 2H, CH_2) ， δ2.68 (m, 4H, NCH_2); ^{13}C NMR(100MHz, $CDCl_3$) δ180.89, 175.10, 142.48, 139.95, 133.08, 131.49, 130.28, 129.84, 128.97, 128.80, 127.98, 127.72, 127.63, 127.07, 127.04, 59.26, 58.05, 52.55, 52.12, 48.71, 48.38, 43.05; HRMS: $C_{22}H_{23}N_3O_2S$, calc.: 394.1511[M+H$^+$]; Found: 394.1580. The crystal structure of Comp8 was solved by X-ray diffraction with direct methods using SHELXS-97.

Authors' contributions

S.Y.Z., H.C., J.N.L and J.Y.L. conceived the project. J.Y.L. and Z.T. designed the experiment. J.Y.L., Z.T., N.Z., X.Y.L. and C.Y.L. performed the experiments and prepared the manuscript. H.C. and S.Y.Z. supervised the study and contributed reagents/materials. All authors contributed to data analysis.

REFERENCES

1. Day CL, Smits C, Fan FC, Lee EF, Fairlie WD, Hinds MG. Structure of the BH3 Domains from the p53-Inducible BH3-Only Proteins Noxa and Puma in Complex with Mcl-1. J Mol Biol. 2008; 380:958–971.
2. Jones G, Willett P, Glen RC, Leach AR, Taylor R. Development and validation of a genetic algorithm for flexible docking. J Mol Biol. 1997; 267:727.
3. Marvin 15. 10.12.0, 2015, ChemAxon (http://www.chemaxon.com).
4. Friberg A, Vigil D, Zhao B, Daniels RN, Burke JP, Garcia-Barrantes PM, Camper D, Chauder BA, Lee T, Olejniczak ET, Fesik SW. Discovery of Potent Myeloid Cell Leukemia 1 (Mcl-1) Inhibitors Using Fragment-Based Methods and Structure-Based Design. J Med Chem. 2013; 56:15–30.
5. Case DA, Betz RM, Botello-Smith W, Cerutti DS, Cheatham TE, III, Darden TA, Duke RE, Giese TJ, Gohlke H, Goetz AW, Homeyer N, Izadi S, et al. AMBER 12, University of California, San Francisco. 2012.
6. Hornak V, Abel R, Okur A, Strockbine B, Roitberg A, Simmerling C. Comparison of multiple Amber force fields and development of improved protein backbone parameters. Proteins: Structure, Function, and Bioinformatics. 2006; 65:712–725.

7. Wang J, Wolf RM, Caldwell JW, Kollman PA, Case DA. Development and testing of a general amber force field. J Comput Chem. 2004; 25:1157–1174.
8. Koes DR, Camacho CJ. PocketQuery: protein–protein interaction inhibitor starting points from protein–protein interaction structure. Nucleic Acids Res. 2012; 40:387–392.
9. Korb O, Stutzle T, Exner TE. Empirical scoring functions for advanced protein-ligand docking with PLANTS. J Chem Inf Model. 2009; 49:84–96.
10. Liu J, Yang X, Zhang Y. Characterization of a lambda-cyhalothrin metabolizing glutathione S-transferase CpGSTd1 from *Cydia pomonella* (L.). Appl Microbiol Biotechnol. 2014; 98:8947–8962.
11. Yang X, Liu J, Li X, Chen M, Zhang Y. Key amino acid associated with acephate detoxification by *Cydia pomonella* carboxylesterase based on molecular dynamics with alanine scanning and site-directed mutagenesis. J Chem Inf Model. 2014; 54:1356–1370.
12. Tian Z, Liu J, Zhang Y. Structural insights into *Cydia pomonella* pheromone binding protein 2 mediated prediction of potentially active semiochemicals. Sci Rep. 2016; 6:22336.
13. Frisch MJ, Trucks GW, Schlegel HB, Scuseria GE, Robb MA, Cheeseman JR, Scalmani G, Barone V, Mennucci B, Petersson GA, Nakatsuji H, Caricato M, Li X, et al. Gaussian 09, Revision B.01. Gaussian, Inc, Wallingford CT, 2010.
14. Wang J, Wolf RM, Caldwell JW, Kollman PA, Case DA. Development and testing of a general amber force field. J Comput Chem. 2004; 25:1157–1174.
15. Wang J. How well does a restrained electrostatic potential (RESP) model perform in calculating conformational energies of organic and biological molecules. J Comput Chem. 2000; 21:1049.
16. Jorgensen WL. Theoretical studies of medium effects on conformational equilibria. J Phys Chem. 1983; 87:5304.
17. Metz A, Pfleger C, Kopitz H, Pfeiffer-Marek S, Baringhaus K-H, Gohlke H. Hot spots and transient pockets: Predicting the determinants of small-molecule binding to a protein–protein interface. J Chem Inf Model. 2012; 52:120–133.
18. Oltersdorf T, Elmore SW, Shoemaker AR, Armstrong RC, Augeri DJ, Belli BA, Bruncko M, Deckwerth TL, Dinges J, Hajduk PJ, Joseph MK, Kitada S, Korsmeyer SJ, Kunzer AR, Letai A, Li C, et al. An inhibitor of Bcl-2 family proteins induces regression of solid tumours. Nature. 2005; 435:677–681.
19. Gohlke H, Kiel C, Case DA. Insights into protein–protein binding by binding free energy calculation and free energy decomposition for the Ras–Raf and Ras–RalGDS complexes. J Mol Biol. 2003; 330:891–913.
20. Raha K, van der Vaart AJ, Riley KE, Peters MB, Westerhoff LM, Kim H, Merz KM. Pairwise decomposition of residue interaction energies using semiempirical quantum mechanical methods in studies of protein−ligand interaction. J Am Chem Soc. 2005; 127:6583–6594.
21. Kollman PA, Massova I, Reyes C, Kuhn B, Huo S, Chong L, Lee M, Lee T, Duan Y, Wang W, Donini O, Cieplak P, Srinivasan J, Case DA, Cheatham TE. Calculating structures and free energies of complex molecules: combining molecular mechanics and continuum models. Accounts Chem Res. 2000; 33:889–897.
22. Miller BR, McGee TD, Swails JM, Homeyer N, Gohlke H, Roitberg AE. MMPBSA.py: An efficient program for end-state free energy calculations. J Chemi Theory Comput. 2012; 8:3314–3321.
23. Yang X, Liu J, Li X, Chen M, Zhang Y. Key amino acid associated with acephate detoxification by *Cydia pomonella* carboxylesterate based on molecular dynamics with alanine scanning and site-directed mutagenesis. J Chem Inf Model. 2014; 54:1356–1370.
24. Liu J, Chen X, Zhang Y. Insights into the key interactions between human protein phosphatase 5 and cantharidin using molecular dynamics and site-directed mutagenesis bioassays. Sci Rep. 2015; 5:12305.
25. Hawkins PCD, Skillman AG, Warren GL, Ellingson BA, Stahl MT. Conformer generation with OMEGA: Algorithm and validation using high quality structures from the protein databank and cambridge structural database. J Chem Inf Model. 2010; 50:572–584.
26. Hawkins PCD, Nicholls A. Conformer generation with OMEGA: Learning from the data set and the analysis of failures. J Chem Inf Model. 2012; 52:2919–2936.
27. Wolber G, Langer T. LigandScout: 3D pharmacophores derived from protein-bound ligands and their use as virtual screening filters. J Chem Inf Model. 2005; 45:160–169.
28. Gabriela M, Mei L, Nicki Z, Ahmet B, Lin Z, Michael E, Joel SG, Jian Y, Ivet B. Development of small-molecule PUMA inhibitors for mitigating radiation-induced cell death. Curr Top Med Chem. 2011; 11:281–290.
29. Huang N, Shoichet BK, Irwin JJ. Benchmarking sets for molecular docking. J Med Chem. 2006; 49:6789–6801.
30. Mysinger MM, Carchia M, Irwin JJ, Shoichet BK. Directory of useful decoys, enhanced (DUD-E): Better ligands and decoys for better benchmarking. J Med Chem. 2012; 55:6582–6594.
31. Triballeau N, Acher F, Brabet I, Pin JP, Bertrand HO. Virtual screening workflow development guided by the "Receiver Operating Characteristic" curve approach. application to high-throughput docking on metabotropic glutamate receptor subtype 4. J Med Chem. 2005; 48:2534–2547.
32. Krautscheid Y, Senning CJÅ, Sartori SB, Singewald N, Schuster D, Stuppner H. Pharmacophore modeling, virtual screening, and *in vitro* testing reveal haloperidol, eprazinone, and fenbutrazate as neurokinin receptors ligands. J Chem Inf Model. 2014; 54:1747–1757.
33. Friberg A, Vigil D, Zhao B, Daniels RN, Burke JP, Garcia-Barrantes PM, Camper D, Chauder BA, Lee T, Olejniczak ET. Discovery of potent myeloid cell leukemia 1 (Mcl-1) inhibitors using fragment-based methods and structure-based design. J Med Chem. 2012; 56:15–30.

34. Ho SN, Hunt HD, Horton RM, Pullen JK, Pease LR. Site-directed mutagenesis by overlap extension using the polymerase chain reaction. Gene. 1989; 77:51–59.

35. Yu J, Wang Z, Kinzler KW, Vogelstein B, Zhang L. PUMA mediates the apoptotic response to p53 in colorectal cancer cells. P Natl Acad Sci USA. 2003; 100:1931–1936.

36. Mustata G, Li M, Zevola N, Bakan A, Zhang L, Epperly M, S Greenberger J, Yu J, Bahar I. Development of small-molecule PUMA inhibitors for mitigating radiation-induced cell death. Curr Top Med Chem. 2011; 11:281–290.

37. Zhong H, Simons JW. Direct comparison of GAPDH, β-actin, cyclophilin, and 28S rRNA as internal standards for quantifying RNA levels under hypoxia. Biochem Biophys Res Commun. 1999; 259:523–526.

Supplementary Table 1: Decomposition of binding free energy on a pairwise per-residue level[a]

Resid 1-Resid 2	van der Waals	Electrostatic	Polar Solvation	Total
**Mcl-1-PUMA**				
L141-F251	−0.89	−0.09	0.013	-0.97 ± 0.27
R142-D237	0.08	−42.52	33.01	-9.43 ± 3.28
R143-D237	−0.01	−12.97	12.78	-0.20 ± 0.02
I144-H205	−1.45	−0.75	0.33	-1.87 ± 0.37
A145-G243	−0.75	−1.17	0.13	-1.79 ± 0.30
D146-R244	−0.16	−19.88	19.26	-0.79 ± 0.12
D147-H205	−0.43	−31.61	28.83	-3.22 ± 1.28
L148-G243	−0.75	0.03	0.063	-0.65 ± 0.25
N149-N241	−0.89	−0.07	−0.46	-1.41 ± 0.28
**Mcl-1-Comp8**				
Comp8-F251	−0.95	−0.22	0.03	-1.14 ± 0.28
Comp8-D237	−0.25	1.56	−1.56	-0.25 ± 0.13
Comp8-H205	−1.10	0.26	−0.21	-1.05 ± 0.31
Comp8-R244	−2.99	−6.10	4.95	-4.14 ± 0.83
Comp8-N241	−0.70	−2.24	0.74	-2.20 ± 0.45

[a]Energies shown as contributions from van der Waals energy, electrostatic energy, polar solvation energy and the sum of them (Total) for Mcl-1-PUMA and Mcl-1- Comp8 complexes. All values are given in kcal/mol.

Supplementary Table 2: The determination of the major protonation form of Comp 8 at a specified pH. See_Supplementary_Table_2

Supplementary Table 3: Pharmacophore-Fit Score obtained through virtual screen and the extracted 8 compounds

Index	Structure	ID number	Mol weight	Pharmacophore-Fit Score
Comp1	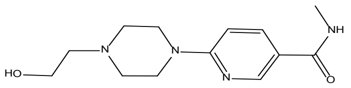	AJ-333/25006049	264.33 Da	62.63
Comp2	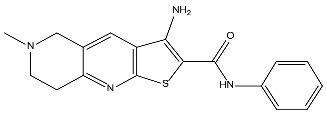	AN-848/40160474	338.43 Da	61.17
Comp3	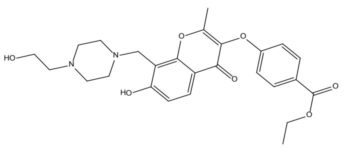	AO-022/42422254	482.53 Da	60.13
Comp4	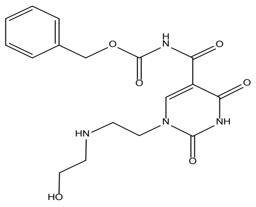	AJ-030/12105275	376.37 Da	59.85
Comp5	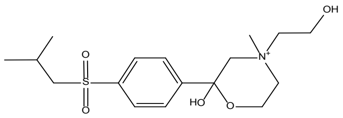	AE-641/30114058	358.48 Da	59.82
Comp6	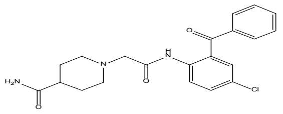	AJ-292/40706371	399.88 Da	59.05
Comp7	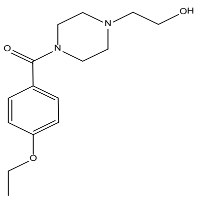	AO-854/43471973	278.35 Da	53.42
Comp8	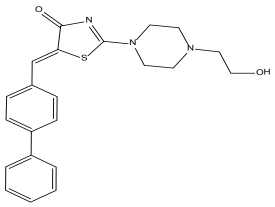	AO-081/41965592	393.51 Da	52.68

Supplementary Table 4: The effects of each residue mutation on the binding between Mcl-1 and the fluorescence probe

Proteins	Mcl-1WT	Mcl-1H205A	Mcl-1D237A	Mcl-1N241A	Mcl-1R244A	Mcl-1F251A
K_i[a]	2.17 ± 0.24	2.35 ± 0.15	2.28 ± 0.09	2.04 ± 0.11	1.98 ± 0.21	2.13 ± 0.06

[a]All values (mean ± SD) are given in kcal/mol.

Supplementary Table 5: Cluster analysis of Mcl-1-PUMA and Mcl-1-Comp8 complexes based on the MD simulations trajectories[a]

System	Cluster	Occurrence [%]	RMSD [7.1] To X-ray/Docking	Primer name	Primer sequence
Mcl-1-PUMA	1	12.6	1.42	Primers for Mcl-1	5′-GCAAGCTTCTAGCCACCTTC TAGGTCCT-3′
	2	34.0	1.52	Mcl-1F	
	3	9.5	1.50	Mcl-1R	5′-GGCGTGCAGCGCAA CGCAGAGACG-3′
	4	21.3	1.58	Primers for mutation	5′-TTGGAAGGCCGTCTCTGCGTTGCG-3′
	5	22.6	1.91	Mcl-1H205F	5′-ATCCATGTTTTCAGCGCAGGCGTA-3′
Mcl-1-Comp8				Mcl-1H205R	5′-CCAGTTTGTTACGCCTGCGCTGAA-3′
	1	10.5	0.88	Mcl-1D237F	5′-AGCGACGGCGTAACAGCATGGGGC-3′
	2	18.1	0.83	Mcl-1N241F	5′-CACAATCCTGCCCCATGCTGTTAC-3′
	3	21.7	1.23	Mcl-1N241R	5′-GTAACAAACTGGGGCGCAATTGTG-3′
	4	5.3	1.51	Mcl-1R244F	5′-AATGAGAGTCACAATTGCGCCCCA-3′
	5	44.4	1.27	Mcl-1R244R	5′-GTGACTCTCATTTCTGCAGGTGCC-3′
				Mcl-1F251F	5′-AGCCACGCAGGCACCTGCAGAAAT-3′
				Mcl-1F251R	

[a]The five structural clusters for two complexes appear consecutively during the 50 ns MD simulations. RMSD values between the cluster and the X-ray structure or the docking structure are typically below 1.5 Å, F: Forward primer; R: Reverse primer

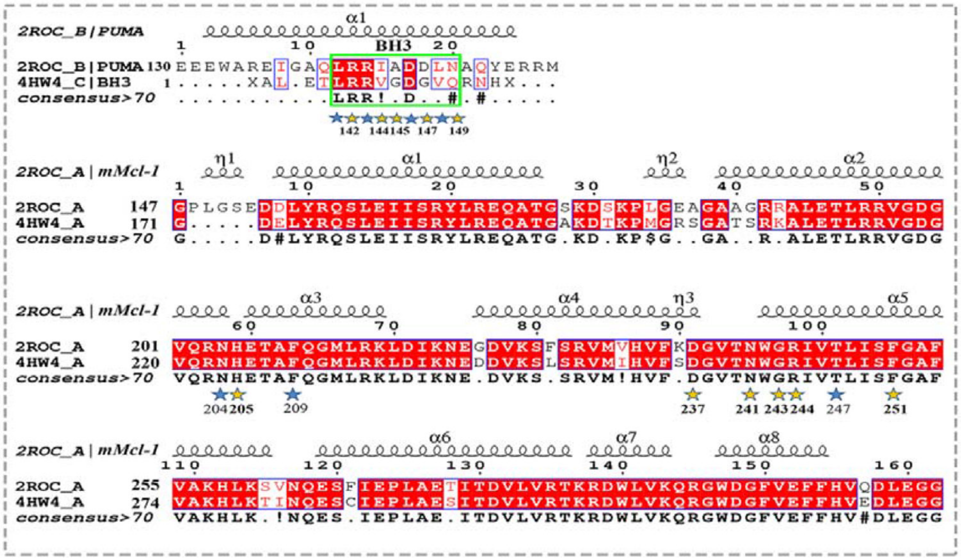

Supplementary Figure 1: The amino acid sequence alignment between mMcl-1 (2ROC_A) and hMcl-1 (4HW4_A). mMcl-1 and hMcl-1 are the Mcl-1 from mouse and human, respectively.

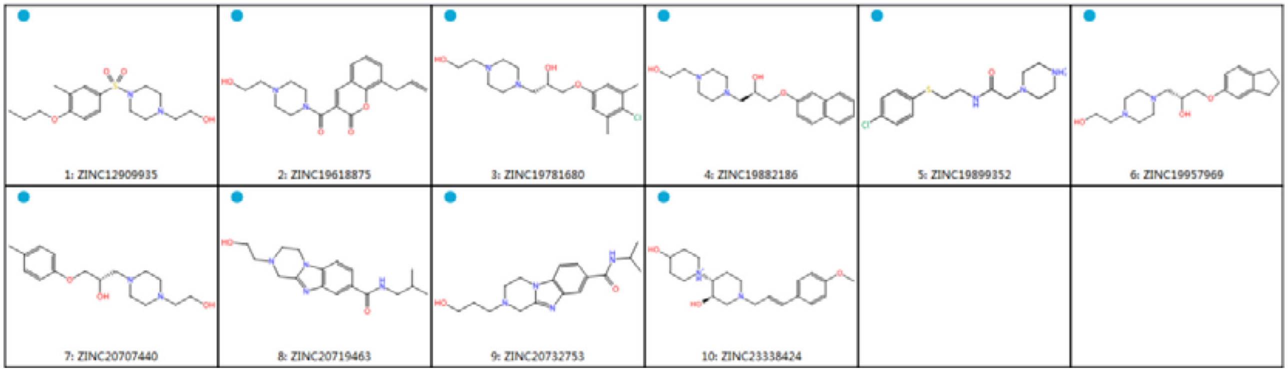

Supplementary Figure 2: The structures of 10 known compounds obviously suppressed PUMA-mediated apoptosis.

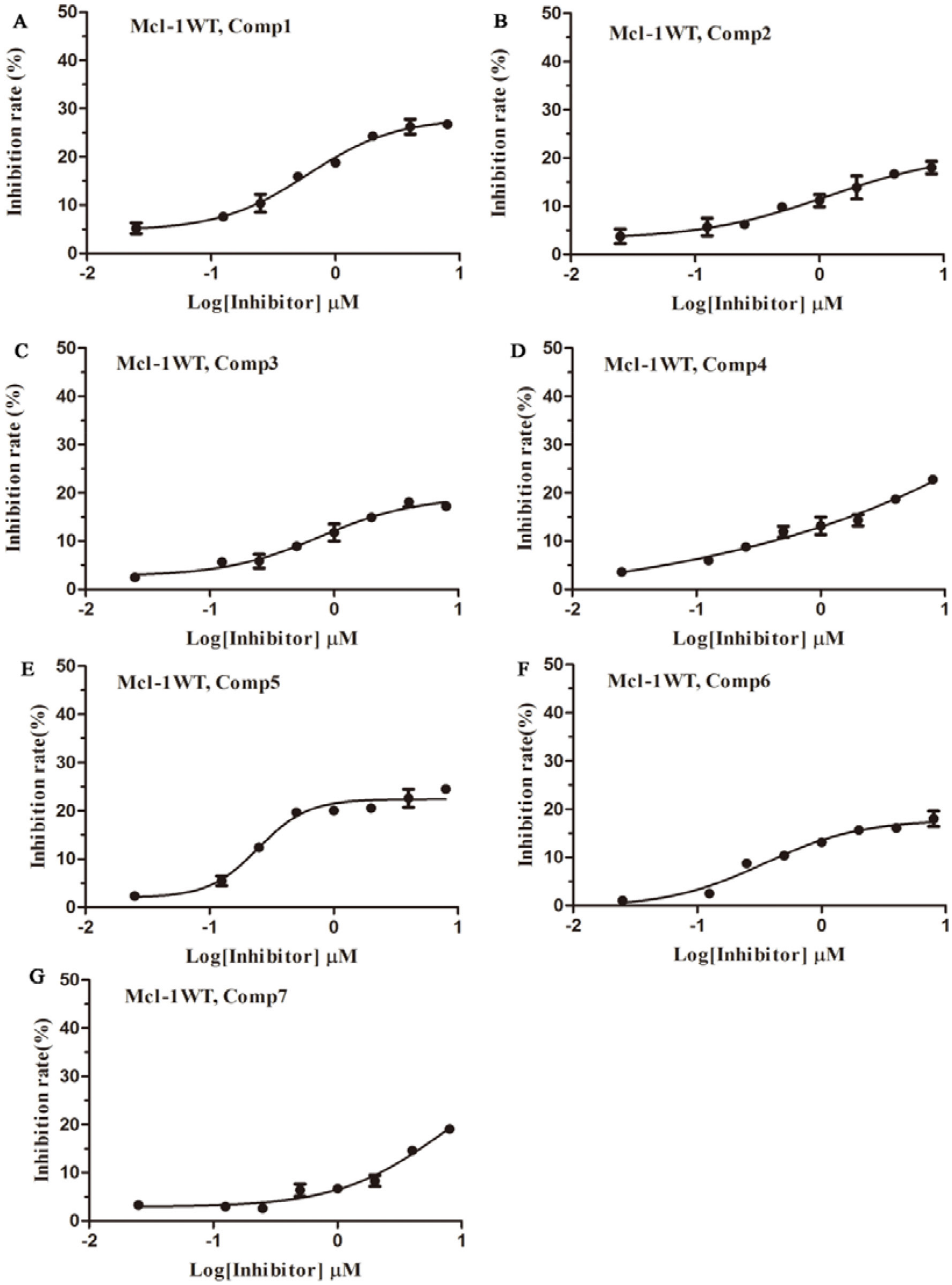

Supplementary Figure 3: The inhibitory effects of (**A–G**) Comp1~Comp7 on wildtype Mcl-1. Mcl-1WT: wildtype Mcl-1; Comp1~Comp7: compound 1~compound 7.

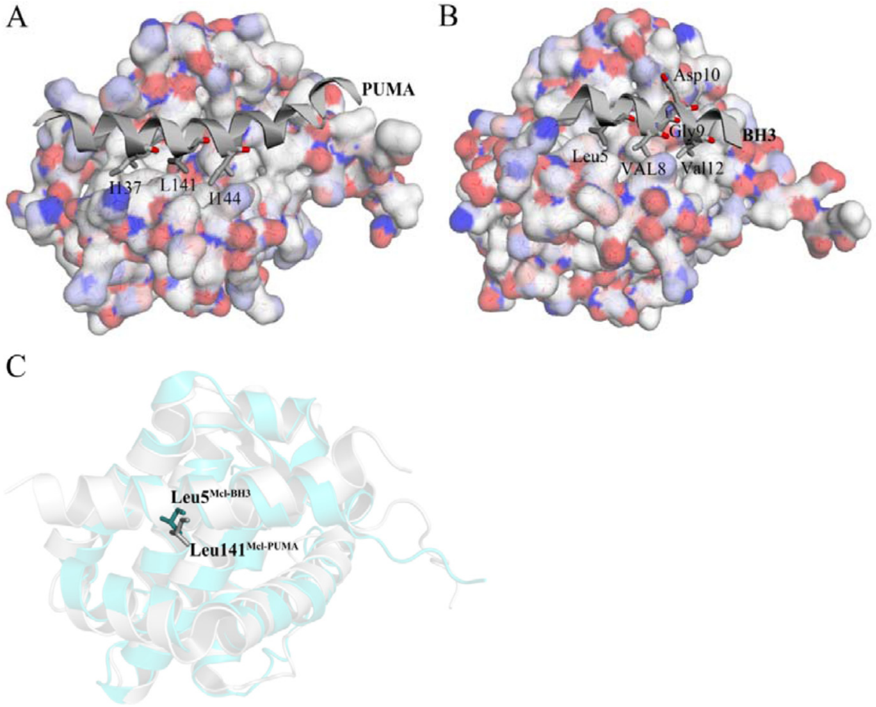

Supplementary Figure 4: The cluster analysis of the interface residues located on (**A**) the crystal structure of Mcl-1 bound to PUMA BH3 (PDB ID, 2ROC) and (**B**) the crystal structure of Mcl-1 bound to its own 16-mer BH3-peptide (PDB ID, 4HW4); (**C**) The structure superimposition of Mcl-1-PUMA and Mcl-1-BH3 complexes

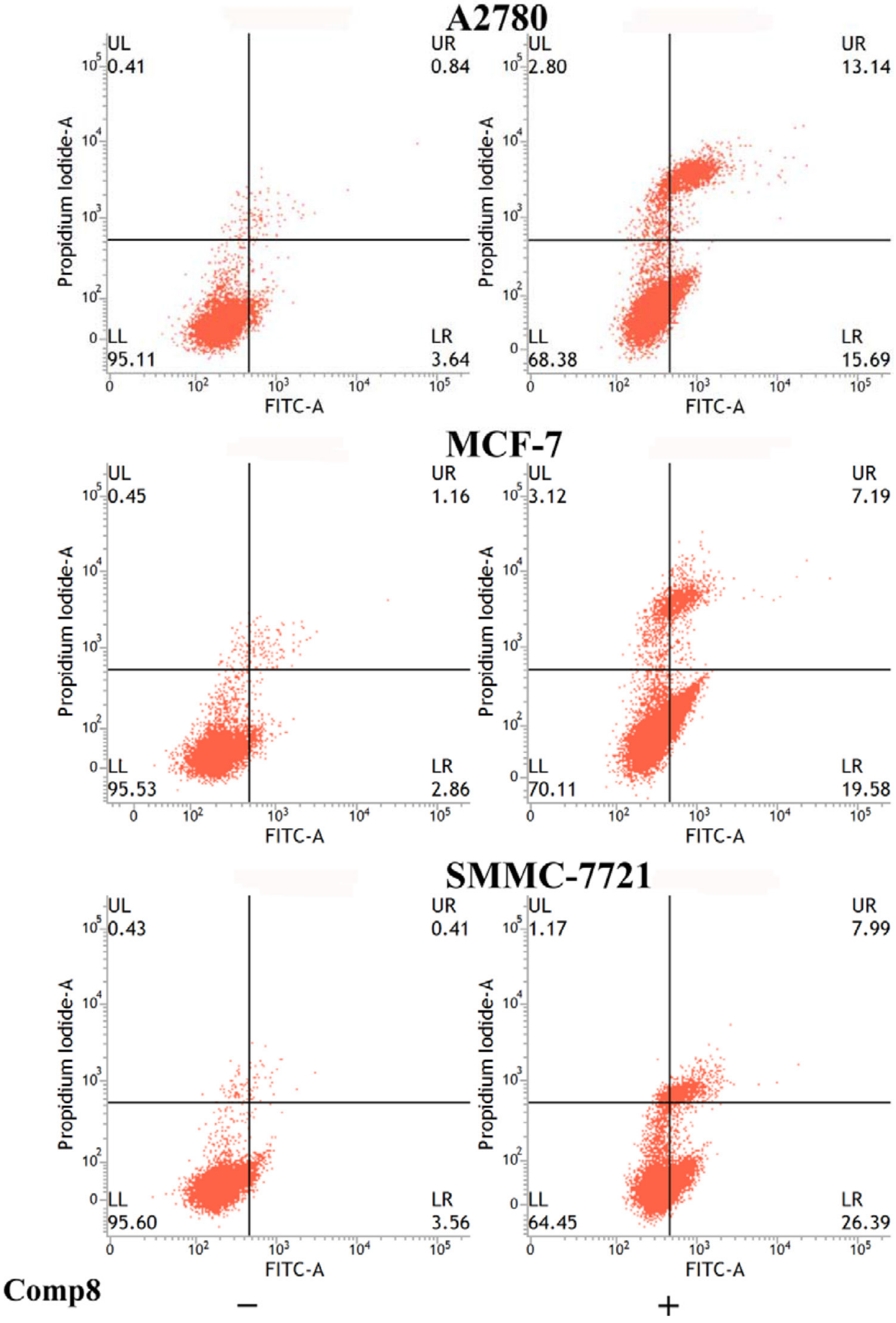

Supplementary Figure 5: The flow cytometry analysis of apoptosis in A2780, MCF-7 and SMMC-7721 cells after treatment with Comp8. “-” means treatment without Comp8, “+” means treatment with Comp8

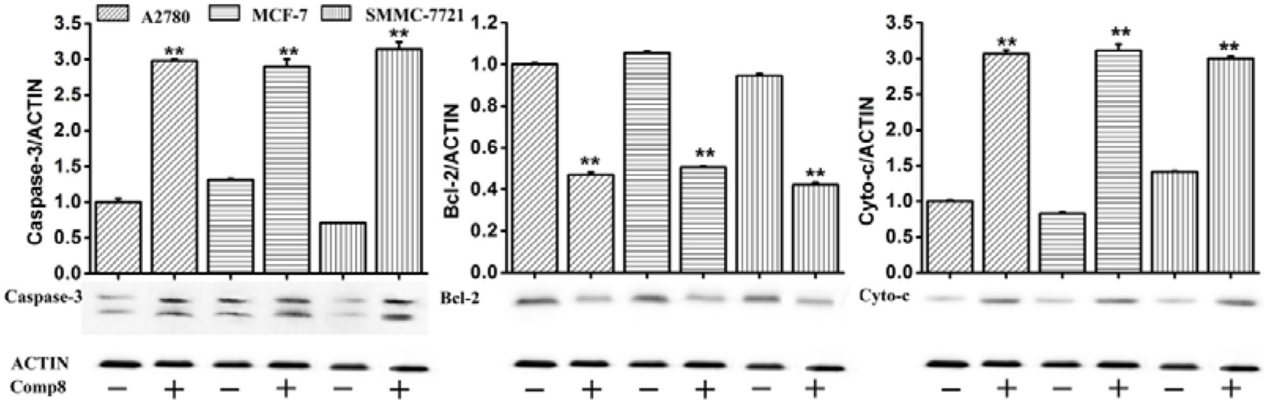

Supplementary Figure 6: Western blot analysis of three apoptotic marker proteins (Caspase-3, Bcl-2 and Cyto-c) in A2780, MCF-7 and SMMC-7721 cells after treatment with Comp8. "-" means treatment without Comp8, "+" means treatment with Comp8.

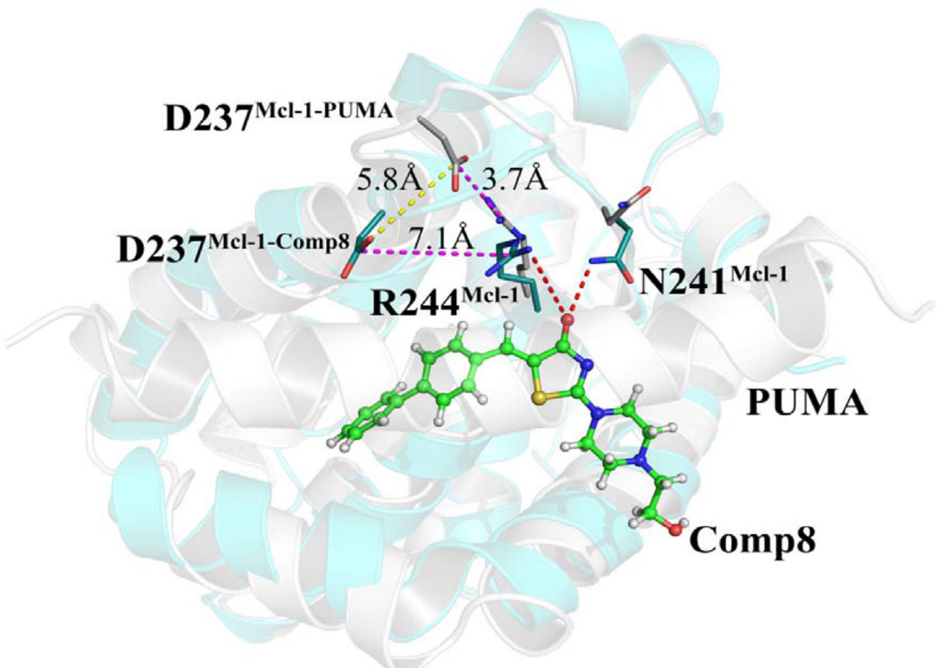

Supplementary Figure 7: The superimposition of the MD representative structures of Mcl-1-PUMA and Mcl-1-Comp8 complexes.

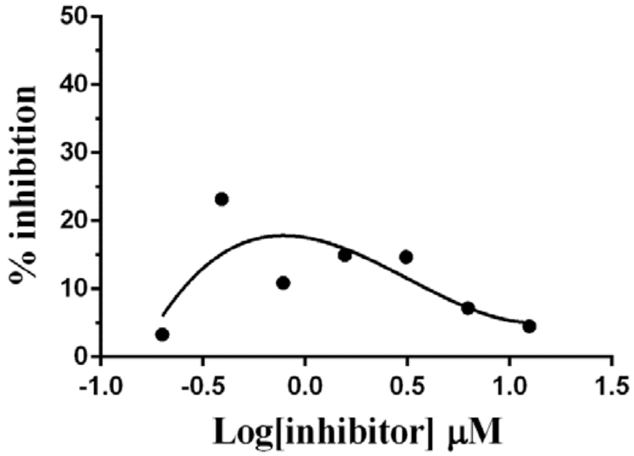

Supplementary Figure 8: The inhibitory effect of Comp8 on HUVECs at low dose.

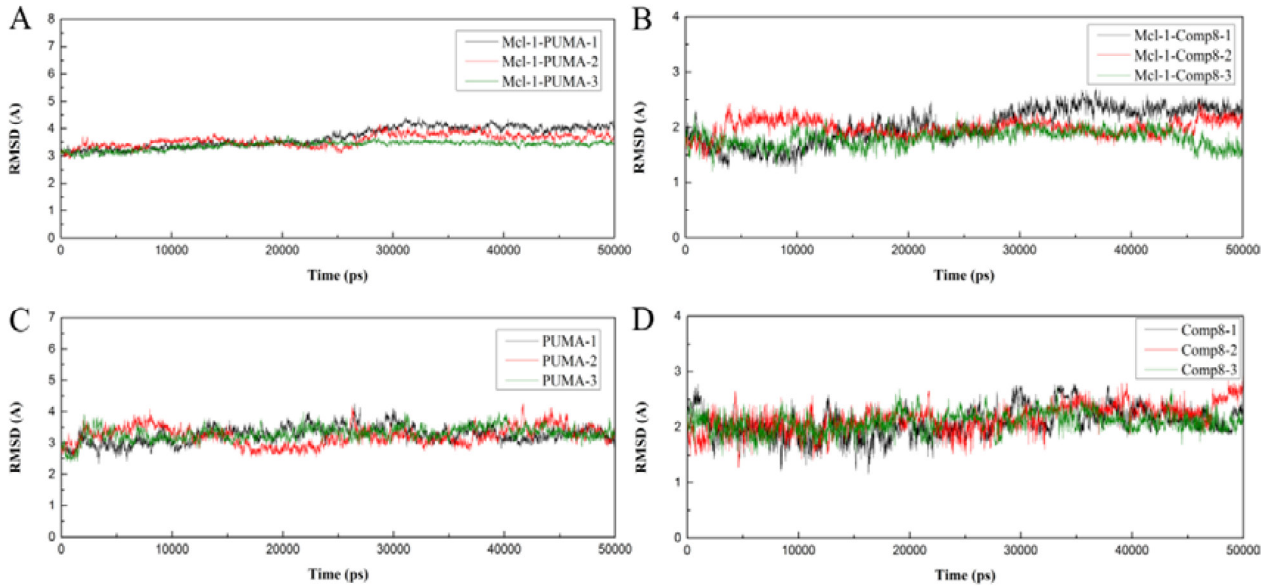

Supplementary Figure 9: The RMSD values for the whole backbone of atoms of Mcl-1-PUMA (**A**) and Mcl-1-Comp8 (**B**) complexes monitored along three individual 50 ns production phase MD simulations. The RMSD of PUMA (**C**) and Comp8 (**D**) relative to the starting structures during three individual 50 ns production phase MD simulations. Comp8: compound 8.

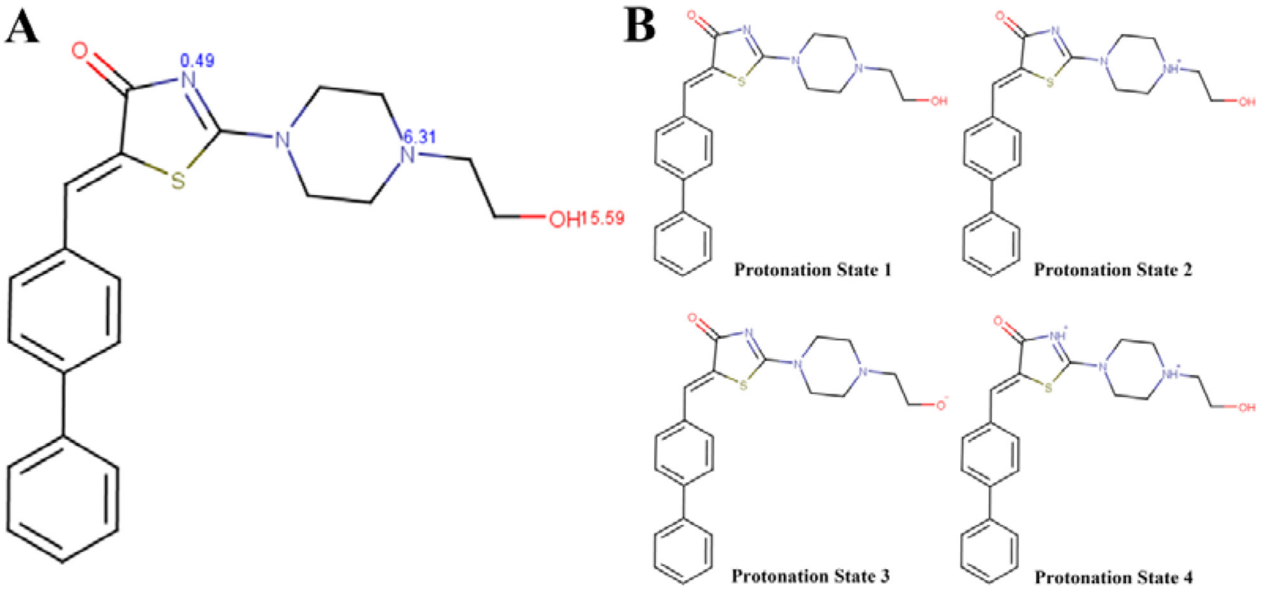

Supplementary Figure 10: (**A**) The prediction of pKa values of donating atoms in Comp 8. (**B**) The major protonation form of Comp 8 at a specified pH.

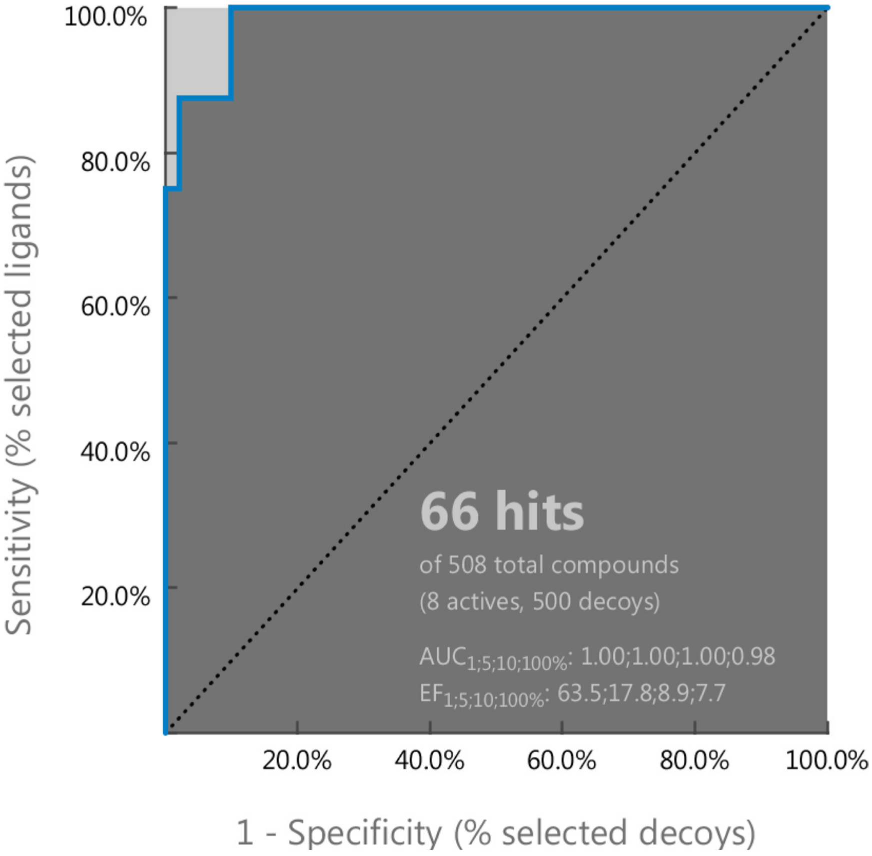

Supplementary Figure 11: ROC curve and AUC resulting from the evaluation of the final pharmacophore model: 66 hits of 508 compounds (8 actives, 500 decoys) AUC 1; 5; 10; 100%: 1.00; 1.00; 1.00; 0.98. EF 1; 5; 10; 100%: 63.5; 17.8; 8.9; 7.7. The ROC curves are plotted using LigandScout 4.09.

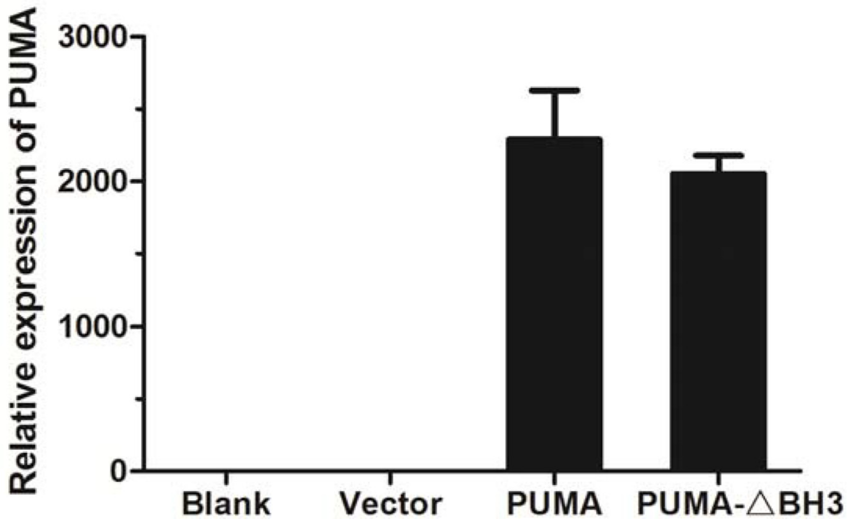

Supplementary Figure 12: The overexpression of PUMA and PUMA-ΔBH3 (PUMA without BH3 domain) in DLD-1 cells.

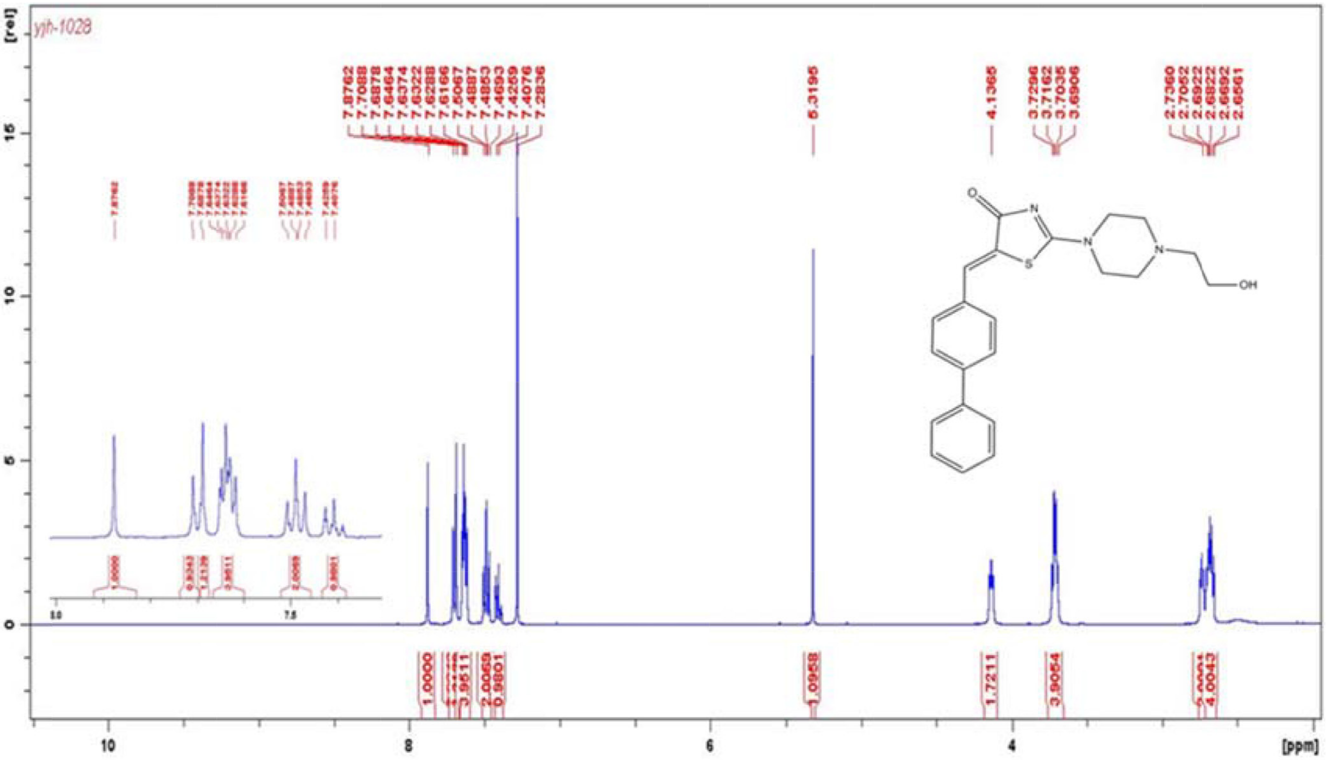

400MHz, ^{1}H NMR in $CDCl_3$

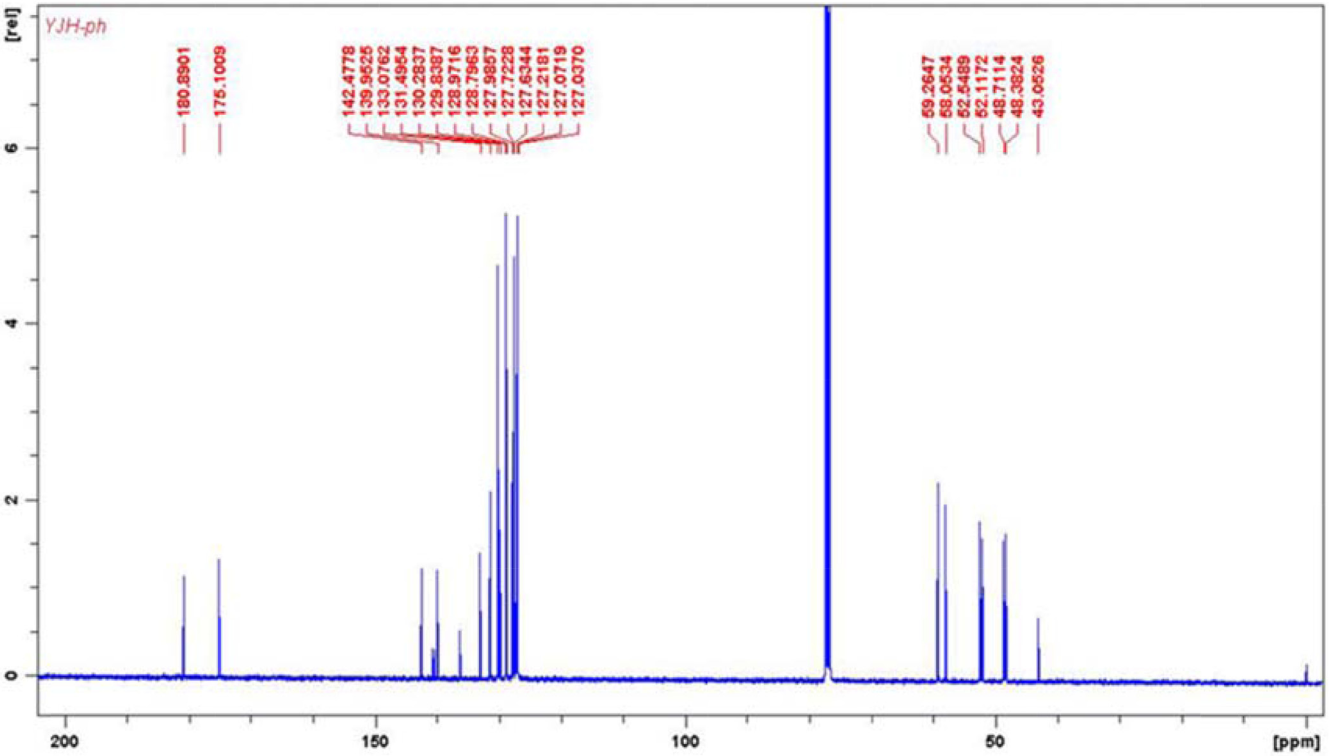

100MHz, ^{13}C NMR in $CDCl_3$

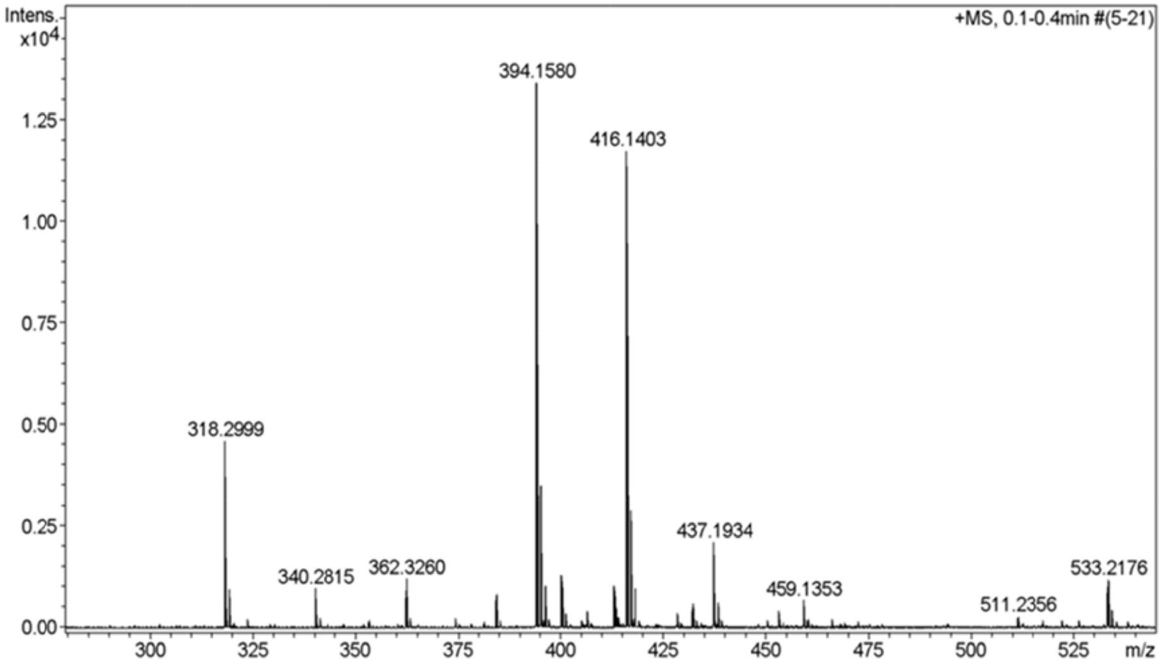

HRMS

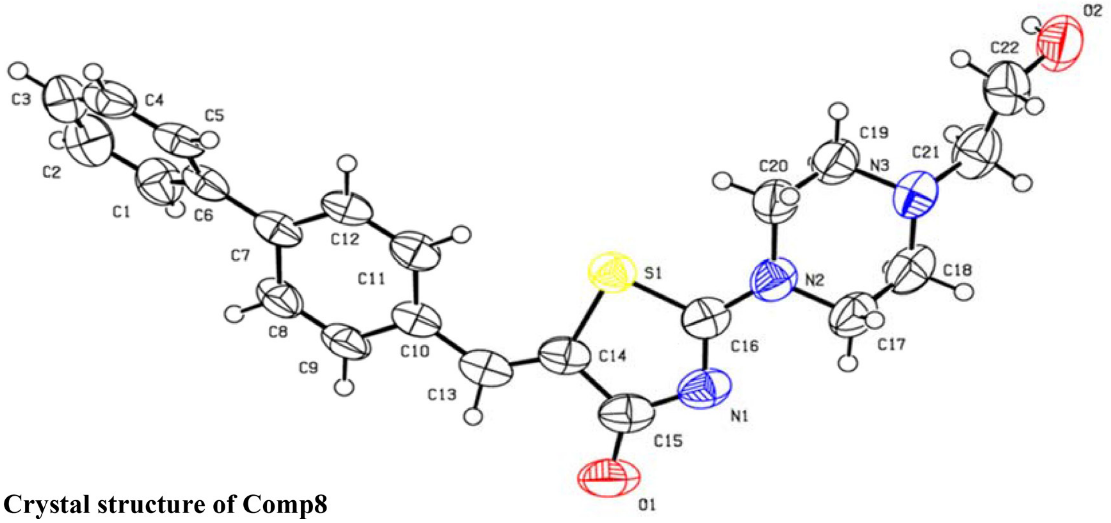

Crystal structure of Comp8

Supplementary Figure 13: Spectral copies of 1H, 13C NMR, HRMS data and crystal structure of Comp8 obtained in this study.

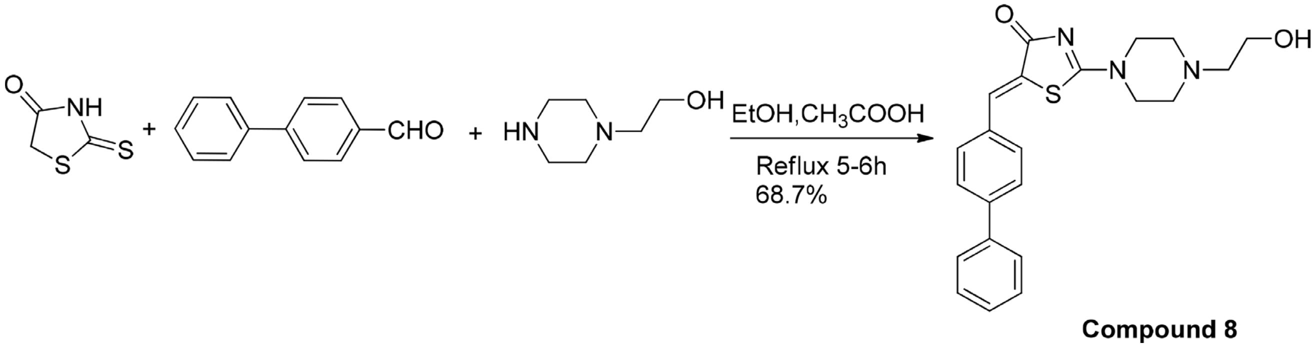

Compound 8

Scheme 1: Preparation of compound 8.
