# Supplementary material for: Targeting the apoptotic Mcl-1-PUMA interface with a dual-acting compound
Source: Oncotarget. 2017 Apr 20;8(33):54236–42. doi: 10.18632/oncotarget.17294 (PMC5589576; doi:10.18632/oncotarget.17294)
Supplement: Supplementary file 2 [file oncotarget-08-54236-s002.pdf]

Supplementary Table 2: The determination of the major protonation form of Comp 8 at a specified pH

pH	%PS1	%PS2	%PS3	%PS4
**0.00**	0.00	24.61	0.00	75.39
**0.20**	0.00	34.09	0.00	65.91
**0.40**	0.00	45.05	0.00	54.95
**0.60**	0.00	56.51	0.00	43.49
**0.80**	0.00	67.31	0.00	32.69
**1.00**	0.00	76.55	0.00	23.45
**1.20**	0.00	83.80	0.00	16.20
**1.40**	0.00	89.13	0.00	10.87
**1.60**	0.00	92.85	0.00	7.15
**1.80**	0.00	95.37	0.00	4.63
**2.00**	0.00	97.02	0.00	2.97
**2.20**	0.01	98.10	0.00	1.90
**2.40**	0.01	98.78	0.00	1.20
**2.60**	0.02	99.22	0.00	0.76
**2.80**	0.03	99.49	0.00	0.48
**3.00**	0.05	99.65	0.00	0.31
**3.20**	0.08	99.73	0.00	0.19
**3.40**	0.12	99.75	0.00	0.12
**3.60**	0.20	99.73	0.00	0.08
**3.80**	0.31	99.64	0.00	0.05
**4.00**	0.49	99.48	0.00	0.03
**4.20**	0.78	99.20	0.00	0.02
**4.40**	1.22	98.76	0.00	0.01
**4.60**	1.93	98.07	0.00	0.01
**4.80**	3.02	96.98	0.00	0.00
**5.00**	4.70	95.30	0.00	0.00
**5.20**	7.25	92.75	0.00	0.00
**5.40**	11.03	88.97	0.00	0.00
**5.60**	16.42	83.58	0.00	0.00
**5.80**	23.74	76.26	0.00	0.00
**6.00**	33.04	66.96	0.00	0.00
**6.20**	43.88	56.12	0.00	0.00
**6.40**	55.34	44.66	0.00	0.00
**6.60**	66.26	33.74	0.00	0.00
**6.80**	75.69	24.31	0.00	0.00
**7.00**	83.15	16.85	0.00	0.00
**7.20**	88.66	11.34	0.00	0.00
**7.40**	92.53	7.47	0.00	0.00
**7.60**	95.16	4.84	0.00	0.00
**7.80**	96.89	3.11	0.00	0.00
**8.00**	98.01	1.99	0.00	0.00
**8.20**	98.74	1.26	0.00	0.00
**8.40**	99.20	0.80	0.00	0.00
**8.60**	99.49	0.51	0.00	0.00
**8.80**	99.68	0.32	0.00	0.00
**9.00**	99.80	0.20	0.00	0.00
**9.20**	99.87	0.13	0.00	0.00
**9.40**	99.92	0.08	0.00	0.00
**9.60**	99.95	0.05	0.00	0.00
**9.80**	99.97	0.03	0.00	0.00
**10.00**	99.98	0.02	0.00	0.00

**10.20**	99.99	0.01	0.00	0.00
**10.40**	99.99	0.01	0.00	0.00
**10.60**	99.99	0.01	0.00	0.00
**10.80**	100.00	0.00	0.00	0.00
**11.00**	100.00	0.00	0.00	0.00
**11.20**	99.99	0.00	0.00	0.00
**11.40**	99.99	0.00	0.01	0.00
**11.60**	99.99	0.00	0.01	0.00
**11.80**	99.98	0.00	0.02	0.00
**12.00**	99.97	0.00	0.03	0.00
**12.20**	99.96	0.00	0.04	0.00
**12.40**	99.94	0.00	0.06	0.00
**12.60**	99.90	0.00	0.10	0.00
**12.80**	99.84	0.00	0.16	0.00
**13.00**	99.75	0.00	0.25	0.00
**13.20**	99.60	0.00	0.40	0.00
**13.40**	99.36	0.00	0.64	0.00
**13.60**	98.99	0.00	1.01	0.00
**13.80**	98.42	0.00	1.58	0.00
**14.00**	97.51	0.00	2.49	0.00

%PS represents the occupancy rate of protonation form of Comp 8
